# Supplementary material for: Multiplexed Optical Sensors in Arrayed Islands of Cells for multimodal recordings of cellular physiology
Source: Nat Commun. 2020 Aug 4;11:3881. doi: 10.1038/s41467-020-17607-5 (PMC7403318; doi:10.1038/s41467-020-17607-5)
Supplement: Supplementary file 1 — Supplementary Information [file 41467_2020_17607_MOESM1_ESM.docx]

**Supplementary** **Information**

**Multiplexed Optical Sensors in Arrayed Islands of Cells for multimodal recordings of cellular physiology**

Christopher A. Werley^1,2^, Stefano Boccardo^1,3^, Alessandra Rigamonti^4^, Emil M. Hansson^4,5^, Adam E. Cohen^1,6 *^

^1^ Department of Chemistry and Chemical Biology, Harvard University, Cambridge, MA 02138

^2^ Current Affiliation: Q-State Biosciences, Cambridge, MA

^3^ Current Affiliation: Nobel Biocare AG, Kloten, Switzerland

^4^ Integrated Cardio Metabolic Centre, Department of Medicine Huddinge, Karolinska Institute, Huddinge, Sweden

^5^ Department of Stem Cell and Regenerative Biology, Harvard University, Cambridge, MA

^6^ Howard Hughes Medical Institute

^*^ Correspondence: [cohen@chemistry.harvard.edu](mailto:cohen@chemistry.harvard.edu)

|  | Subcellular localization | Plasmid number | Supp. fig. | Best observed sensitivity | Bright-ness | pH Hill exponent | pKa | *S*, high pH | *S*, low pH |
| --- | --- | --- | --- | --- | --- | --- | --- | --- | --- |
| QuasAr2 | membrane | pMOS009 | 2 | 1.7 (cardiac AP) | * | - | - | - | - |
| CheRiff | membrane | pMOS010 | 3 | - | - | - | - | - | - |
| GCaMP6F | cytosol | pMOS008 | 4 | 2.5 (cardiac AP) | ** | 1.8 | 7.9 | 6.84 | 0.62 |
| Mitycam | mito. | pMOS028 | 5 | 1.06 (cardiac AP) | **** | 1.0 | 6.2 | 0.74 | 7.46 |
| GCaMPer | ER | pMOS003 | 6 | 0.97 (cardiac AP)  0.5 (5 mM caffeine) | ** | 1.4 | 7.4 | 1.35 | 0.32 |
| ClopHensorN | cytosol | pMOS019 | 7 | 0.6 (0 mM Cl-) | *** | 1.2 | 7.1 | 0.60 | 1.54 |
| superecliptic pHluorin | cytosol | pMOS005 | 8 | 4.5 (pH 8.5/pH 6.5) | **** | 1.8 | 7.5 | 2.37 | 0.08 |
|  | mito. | pMOS011 | 9 | 5.5 (pH 8.5/pH 6.5) | *** |  |  |  |  |
|  | ER | pMOS031 | 10 | 6 (pH 7.4/pH 6.5) | **** |  |  |  |  |
| ratiometric pHluorin | cytosol | pMOS017 | 11 | 1.8 (pH 7.5/pH 6.5) | ** | 1.3 | 7.3 | 1.11 | 0.68 |
|  | mito. | pMOS018 | 12 | 1.3 (pH 7.5/pH 6.5) | ** |  |  |  |  |
|  | ER | pMOS030 | 13 | 1.5 (pH 7.4/pH 6.5) | *** |  |  |  |  |
| Grx1-roGFP2 | cytosol | pMOS014 | 14 | 2.6 (1.3 mM H_2_O_2_) | *** | - | - | - | - |
| mito-roGFP2-Grx1 | mito. | pMOS013 | 15 | 1.8 (1.3 mM H_2_O_2_) | **** | - | - | - | - |
| Grx1-roGFP1-iE_ER_ | ER | pMOS012 | 16 | 0.7 (5 mM DTT) | *** | - | - | - | - |
| roGFP2-Orp1 | cytosol | pMOS016 | 17 | 2.5 (1.3 mM H_2_O_2_) | *** | - | - | - | - |
| mito-roGFP2-Orp1 | mito | pMOS015 | 18 | 3.1 (2 mM H_2_O_2_) | *** | - | - | - | - |
| Peredox | cytosol | pMOS023 | 19 | 2.5 (10 mM lactate:  0 mM pyruvate) | *** | - | - | - | - |
| PercevalHR | cytosol | pMOS007 | 20 | 0.3 (metab. block) | ** | 1.1 | 7.7 | 1.42 | 0.30 |
| FLII^12^Pglu-700μδ6 | cytosol | pMOS020 | 21 | 0.7 (0 mM glucose) | **** | 1.0 | 6.8 | 0.86 | 1.38 |
| upward DAG | cytosol | pMOS024 | 22 | 1.4 (50 μM carbachol) | ** | 1.4 | 7.6 | 2.59 | 0.23 |

**Supplementary Table 1 – Sensor characteristics.** Column **Plasmid number** references the internal plasmid identifier for the lentiviral backbone plasmid. The **best observed sensitivity** is *S*/*S*_0_. If not stated explicitly in the column, *S*_0_ is the sensor response in standard imaging buffer. **Brightness** is a qualitative measure of the relative number of counts collected on the camera when all sensors are excited by a constant illumination intensity. The scale ranges from dimmest (*) to brightest (****). **pH Hill exponent**, **pKa**, ***S* (high pH)**, and ***S* (low pH)** are the fit coefficients characterizing sensor pH response (Fig. 4, Methods). Calculation for the sensor response, *S*, is shown in Table 1.


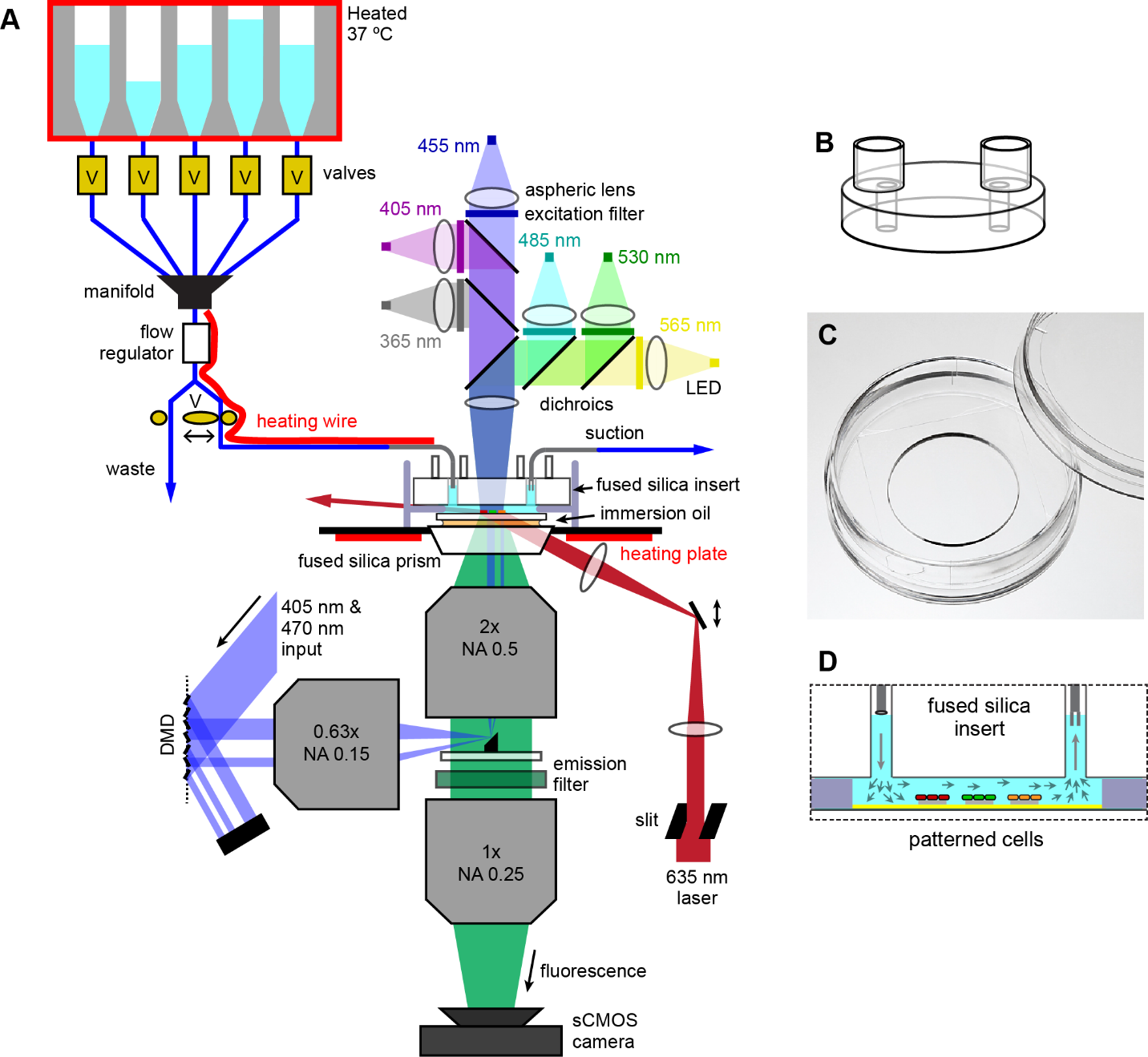


**Supplementary Figure 1 – Perfusion and optical diagram.** (A) The Firefly microscope optical design, described fully in [1], was originally designed for large-area all-optical electrophysiology with Optopatch^2^. The grazing-incidence red laser enables low-background recording of voltage sensor QuasAr. The patterned blue light illumination off the digital micromirror device (DMD) enables targeted stimulation of single cells or regions of a cardiomyocyte syncytium. To image using the various spectral excitation bands required by the different MOSAIC sensors, the microscope is equipped with a 6-color LED system which illuminates from above. The system provides large area, homogeneous illumination, analog control of illumination intensity with sub-millisecond temporal resolution, and simultaneous illumination with multiple colors or rapid switching between colors. Illumination is incident at ~45º (coming out of the plane of the page), to minimize the amount of light that couples into the objective and thus to reduce background autofluorescence from the objective glass.

The sample is heated, and the microscope is equipped with a digitally controlled, fast perfusion system (upper left and inset on lower right). Seven syringe tubes (only 5 are shown) are combined with a manifold, and flow rate is controlled with a metering valve (Swagelok #SS-SS1-VH). The liquid is heated in the syringes, and the temperature is maintained with a heated wire before flowing into the sample chamber. Prior to flow into the sample chamber, the lines are purged by washing media from the selected syringe through the system to waste. In this way, only a small dead volume must flow through the sample and fast media exchange is possible. The perfusion valves, light sources, and camera are synchronized and controlled by a data acquisition (DAQ card; National Instruments #PCIe-6353) and a custom LabView program.

The sample perfusion chamber is formed by inserting a fused silica disk (McMaster #47-834) with two holes ground into it (B) into a glass bottomed dish (C) (Cellvis #D35-20-1.5-N). The chamber height is set by the dish plastic thickness to 1 mm, and the sample achieves quasi-laminar flow (D).


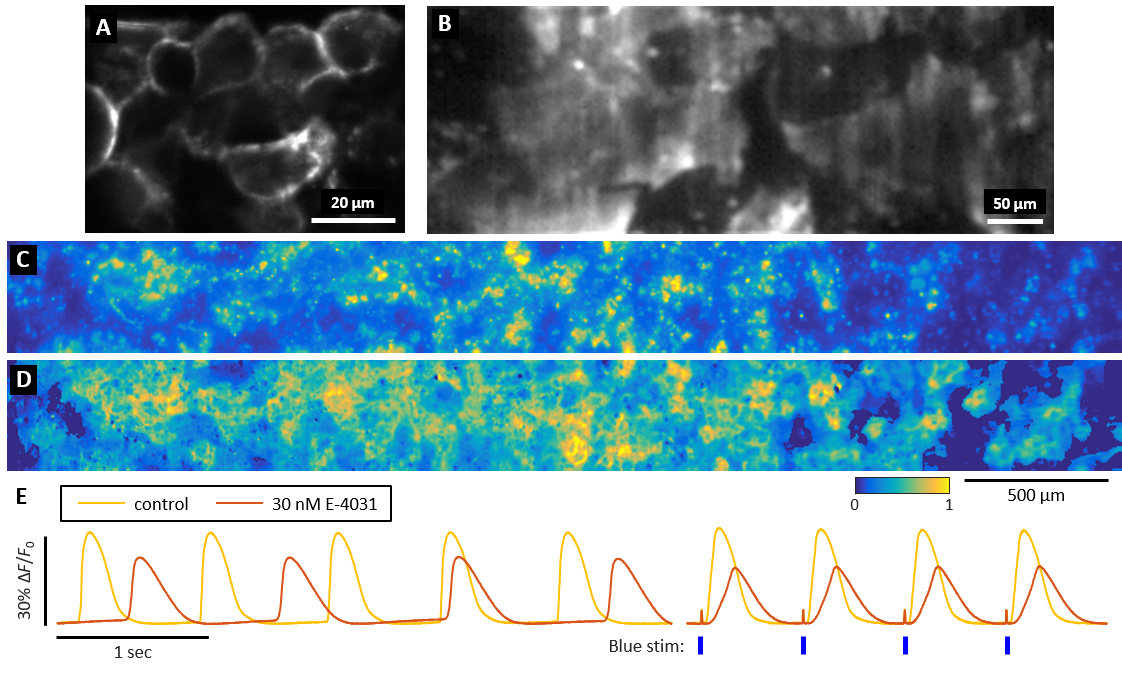


**Supplementary Figure 2 – QuasAr2.** The voltage-sensing protein QuasAr2 is an engineered archaerhodopsin that is excited at 635 nm and has its fluorescence peak at 710 nm. Its ~ 1 ms response time is sufficiently fast to record action potentials in neurons and the far-red fluorescence is compatible with simultaneous channelrhodopsin simulation or recordings with GFP or RFP based sensors. Here it is fused with mOrange2 to show membrane trafficking. QuasAr2 has been used to record action potentials in cultured neurons^2,3^, acute brain slice^4^, cardiomyocytes^5,6^, and spiking HEK cells^7,8^. (A) A high-magnification image of HEK293 cells expressing QuasAr2, collected in the mOrange2 channel. (B) QuasAr2 fluorescence in human iPS-derived cardiomyocytes (CMs). (C) A widefield image of QuasAr2 fluorescence in CMs. (D) Same field of view as (C), showing the sensitivity (Δ*F*/*F*_0_) for each pixel to highlight the membrane-trafficked fluorescent reporter. (E) QuasAr2 fluorescence time trace from these cells during spontaneous beating (left) and during pacing at 1.5 Hz with CheRiff (right). Yellow is the control trace and red is after addition of 30 nM E-4031, a blocker of the HERG potassium channel. The drug changes the spontaneous beat rate, slows the upstroke velocity, and reduces the amplitude. Recordings were made at a 500 Hz frame rate. When calculating the fluorescence trace, pixels were weighted based on their SNR to exclude bright, unresponsive puncta and improve sensitivity.


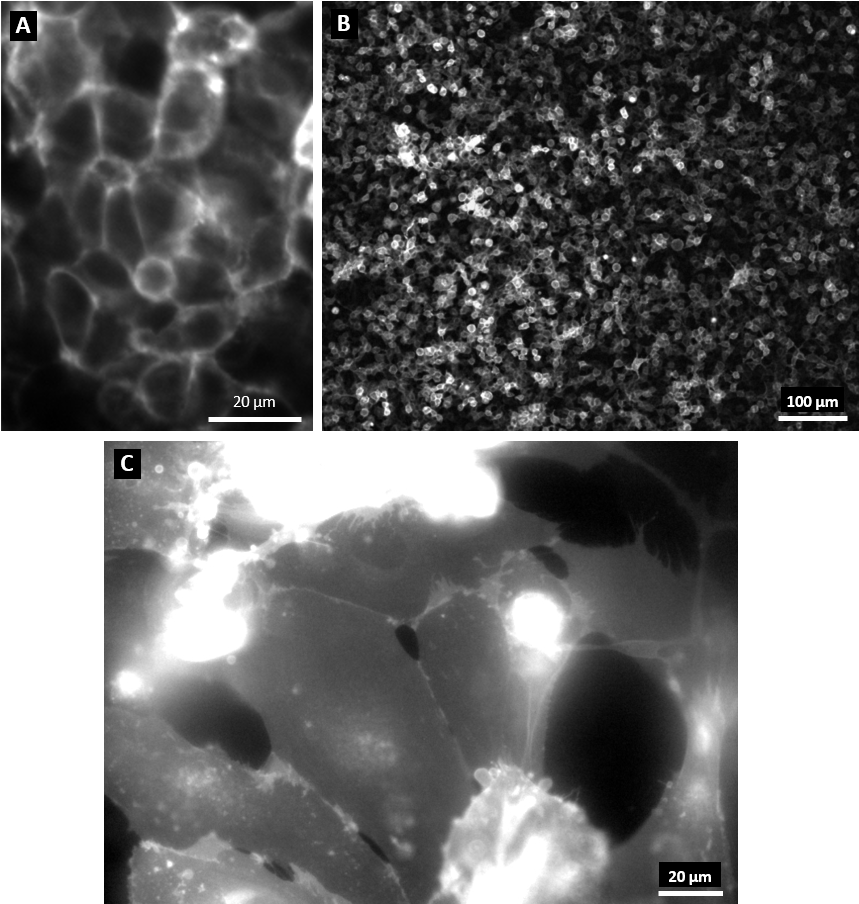


**Supplementary Figure 3 – CheRiff.** CheRiff is a channelrhodopsin, a non-selective light-gated cation channel with activation peak at 470 nm^2^. Here it is fused with EGFP to show membrane trafficking. CheRiff passes more current for the same illumination intensity than ChR2 and has been used to stimulate action potentials in cultured neurons^2,3,9^, acute brain slice^4^, cardiomyocytes^5,6^, and spiking HEK cells^7,8^. Pacing of cardiomyocytes is demonstrated in figure S2. (A) A high-magnification image of HEK293 cells expressing CheRiff. (B) A widefield image of lentivirally transfected HEK293 cells showing efficient transfection. (C) CheRiff fluorescence in human iPS-derived cardiomyocytes showing membrane trafficking.


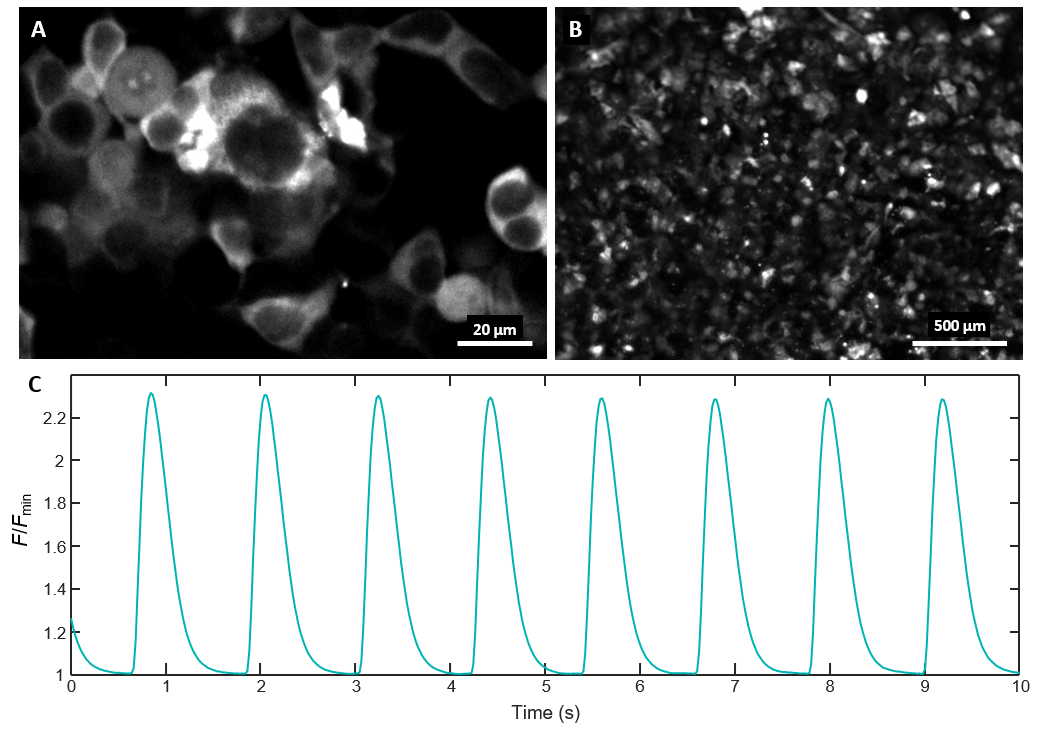


**Supplementary Figure 4 – Cytosolic GCaMP6f.** This reporter is engineered by attaching a circularly permuted green fluorescent protein (cpGFP) fused to the calcium-binding protein calmodulin (CaM) and the associated M13 peptide^10^. Upon Ca^2+^ binding, the CaM and M13 come together and make the sensor bright. One of the most highly optimized fluorescent sensors^11^, the calcium binding constant of GCaMP6f (f for “fast”) is tuned so the sensor is dim at resting cytosolic calcium concentration and becomes much brighter during a neuronal action potential. In ventricular cardiomyocytes, where cytosolic calcium concentration changes from 62 nM to 135 nM during an action potential^12^, the fluorescence can change more than 2-fold. (A) A high-magnification image of HEK293 cells expressing GCaMP6f. (B) A widefield image of human iPS-derived cardiomyocytes (CMs) expressing this reporter and (C) the fluorescence time trace from these cells as they beat spontaneously. The CMs are excited at 485 nm and fluorescence is recorded at 500 Hz in the GFP channel. This sensor has been used extensively to probe pharmacological effects in similar CMs^5,6^.


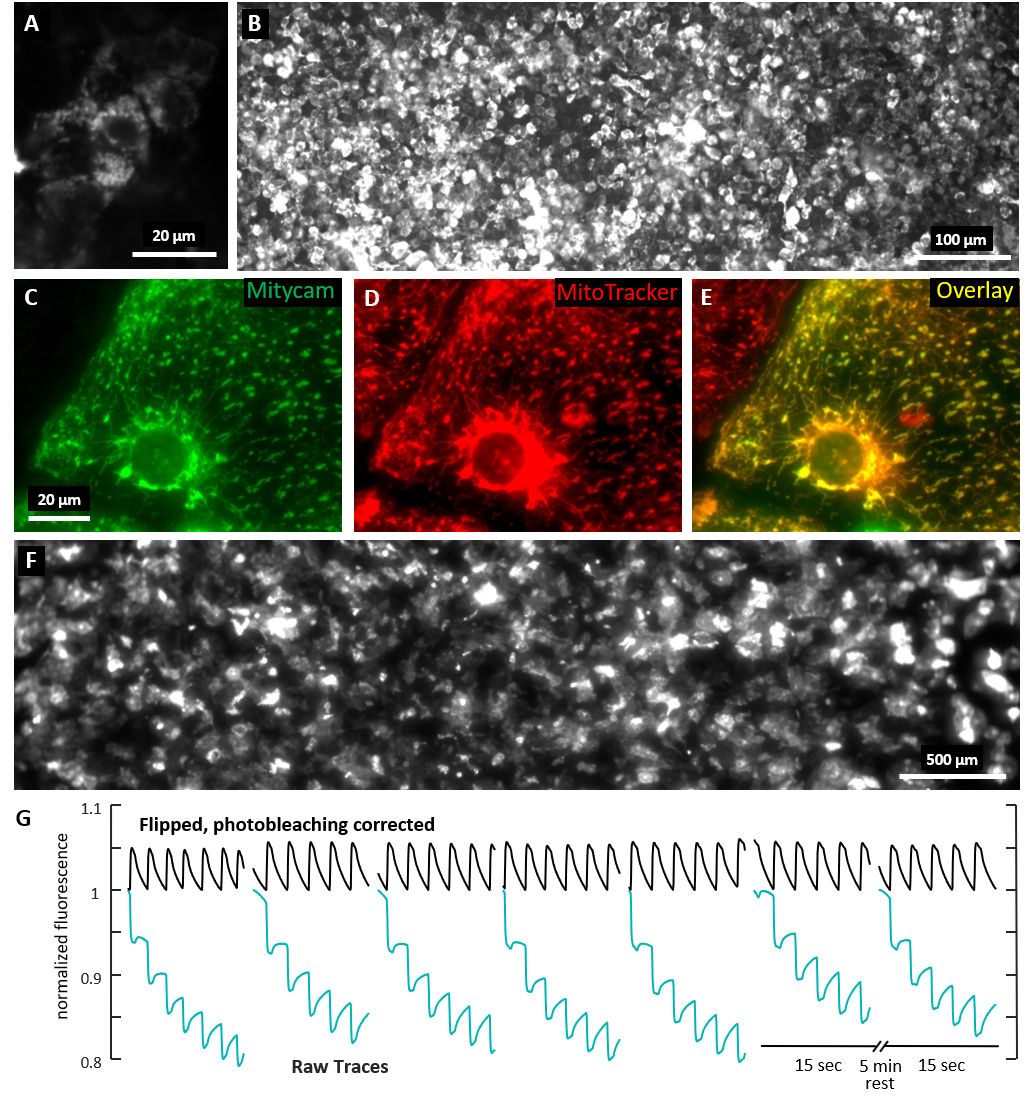


**Supplementary Figure 5 – Mitochondrially-targeted Mitycam.** This calcium reporter is engineered by attaching a circularly permuted yellow fluorescent protein (cpYFP) to the calcium-binding protein calmodulin (CaM) and the associated M13 peptide^13,14^. Upon Ca^2+^ binding, the CaM and M13 come together and distort the chromophore, dimming the cpYFP (inverse sensor). It is targeted to the mitochondria using the pre-sequence of the human cytochrome oxidase subunit IV. The sensor has been used in cardiomyocytes in multiple studies^13–16^. (A) A high-magnification image of HEK293 cells expressing this reporter. (B) A widefield image of HEK293 cells lentivirally transfected with Mitycam. (C) Mitycam, (D) MitoTracker Red FM (ThermoFisher #M22425), and (E) overlay, showing mitochondrial trafficking of the sensor in human iPS-derived cardiomyocytes (CMs). (F) Wide-field image of Mitycam in CMs and (G) fluorescence recordings from these cells as they beat spontaneously. The sensor is excited at 485 nm and fluorescence is recorded at 20 Hz in the GFP channel, with 5 minute rests between 15 sec. recordings. The raw fluorescence (teal) shows the inverse sensitivity to calcium concentration, rapid “photobleaching”, and recovery of photobleaching during the rests. The black recording is inverted and photobleaching corrected.


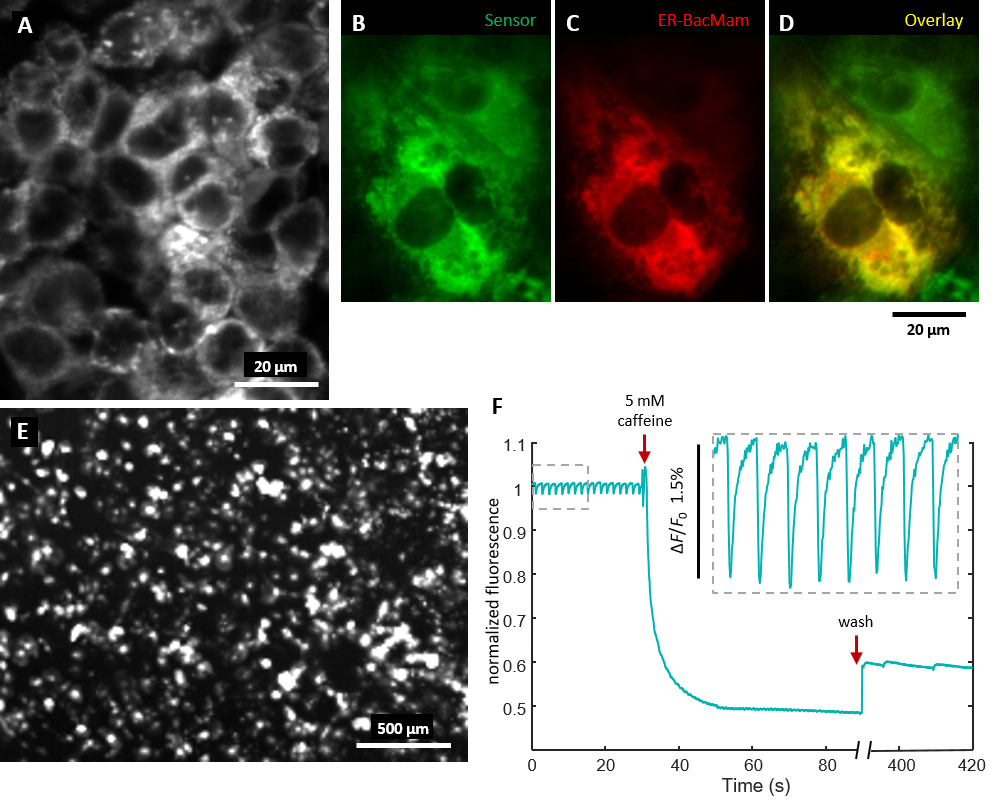


**Supplementary Figure 6 – ER-targeted GCaMPer.** This reporter is an engineered version of GCaMP3, where a circularly permuted green fluorescent protein (cpGFP) fused to the calcium-binding protein calmodulin (CaM) and the associated M13 peptide^17,18^. Upon Ca^2+^ binding, the CaM and M13 come together, and make the sensor bright. It is targeted to the endoplasmic reticulum using the calreticulin signal peptide MLLSVPLLLGLLGLAVA attached to the N-terminus, and the BiP ER retention signal TAEKDEL fused to the C-terminus^18^. Because the typical 500 μM calcium concentration in the ER is 5000x higher than in the cytosol, GCaMPer has its Ca^2+^ binding constant tuned to 400 μM^18^. However, because the relative Ca^2+^ changes only a small percent during a typical cardiomyocyte beat, the Δ*F*/*F*_0_ is only ~1.5% during spontaneous beating. The sensor is excited at 485 nm and fluorescence is collected in the GFP channel. (A) A high-magnification image of HEK293 cells expressing GCaMPer. ER-trafficking in human iPS-derived cardiomyocytes (CMs) demonstrated by (B) CaMPer fluorescence, (C) the same cells with BacMam ER-RFP (ThermoFisher #C10591), an ER marker, and (D) an overlay. (E) Widefield image of GCaMPer fluorescence in cardiomyocytes and (F) fluorescence time trace from these cells as they beat spontaneously. After 30 seconds of recording, caffeine was added to vent the ER calcium to the cytosol. The cardiomyocytes partially recovered after the caffeine was washed out and after a 5 min. rest. Data adapted from [18] with permission.


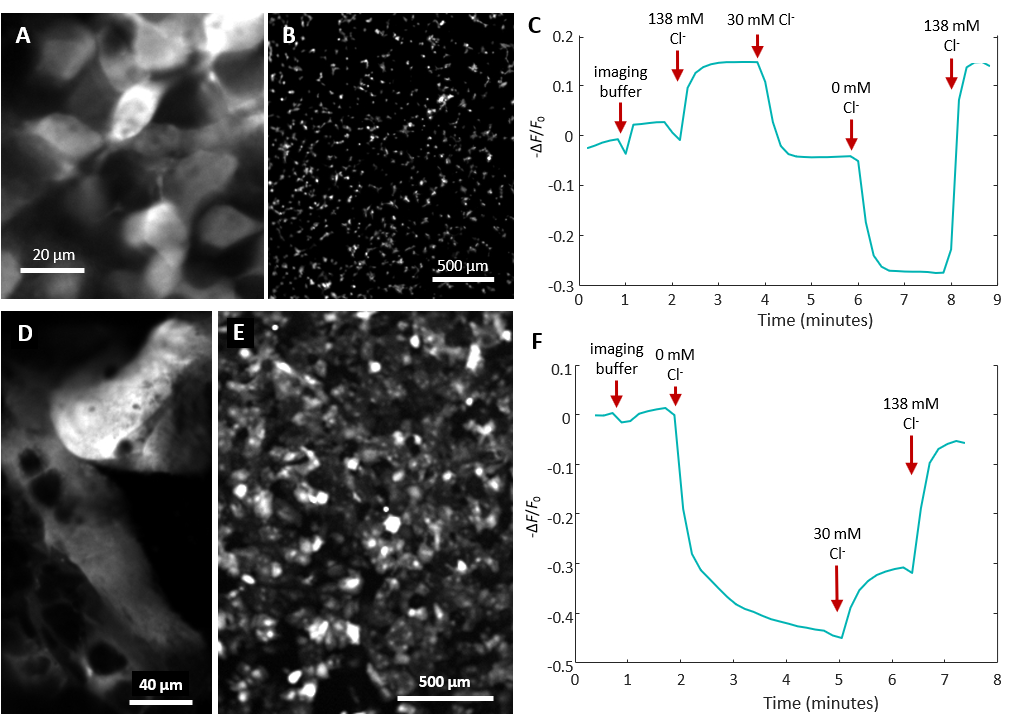


**Supplementary Figure 7 – ClopHensorN.** ClopHensorN is a sensor designed to measure simultaneously [Cl^-^] and pH^19,20^. It is based on E^2^GFP, an EGFP variant engineered to have an anion binding site that responds to chloride and protons^21^. The E^2^GFP is fused to tdTomato, a red-orange fluorescent protein that is pH and [Cl^-^] insensitive, to allow ratiometric correction for variation in expression level^20^. By recording tdTomato fluorescence and E^2^GFP excited at both 488 nm and 458 nm, it is possible to quantitatively measure both [Cl^-^] and pH^19^. In MOSAIC, we know the pH independently from pH sensors, so we can measure [Cl^-^] by collecting E^2^GFP fluorescence under just 488 nm excitation. (A) High magnification image of HEK293 cells expressing ClopHensorN recorded in the GFP channel. (B) Average fluorescence image of HEK293 cells at low magnification and (C) the corresponding time trace during changes in [Cl^-^]. (D) A high-magnification image of ClopHensorN expression in human iPS-derived cardiomyocytes (CMs). (E) The average fluorescence image CMs and (F) their average time trace as [Cl^-^] changes. For both HEK293 cells and CMs, fluorescence generally tracks with [Cl^-^], although the 138 mM changes are far beyond the physiological range. Images were recorded every 10 seconds under illumination at 485 nm and emission in the GFP fluorescence channel. To dissipate electrical and concentration gradients and to enable equilibration of ions [1], we added the K^+^ ionophore valinomycin (5 μM), the protonophore carbonyl cyanide p-chlorophenylhydrazone (CCCP) (5 μM), the K^+^/H^+^ exchanger nigericin (5 μM), and the Cl^−^/OH^−^ exchanger tributyltin chloride (10 μM) in a high-potassium buffer. Different buffers were perfused through the system, holding total osmolarity and all salt concentrations constant except for an equi-molar exchange between chloride and gluconate as the counter-anion: [Cl^-^] + [gluconate^-^] = 138 mM. In addition to the ionophores and exchangers, the buffer was comprised of 20 mM HEPES, 0.6 mM MgSO_4_, 100 mM K^+^ derived from a mixture of potassium gluconate and KCl, and 38 mM Na^+^ derived from a mixture of sodium gluconate and NaCl. Initial imaging buffer did not have ionophores and exchangers. To analyze, any cells that washed away were masked and not included in analysis. The darkest 45% or 30% of pixels were counted as background for HEK293 and CMs respectively, and their time trace was subtracted from the average cell trace.


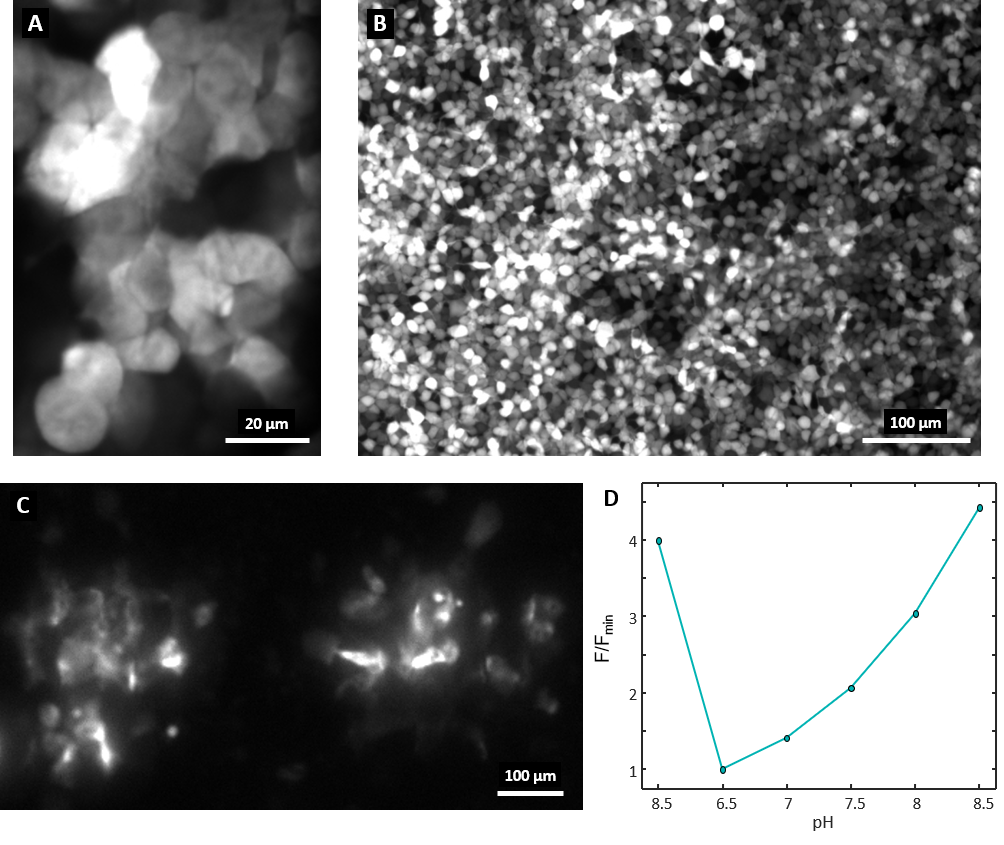


**Supplementary Figure 8 – cytosolic superecliptic pHluorin.** Superecliptic pHluorin, an engineered derivative of EGFP, becomes dimmer at acidic pH due to protonation of the chromophore^22^. Its absorption and emission bands are aligned to those of EGFP. The sensor is bright and highly sensitive, although it is not ratiometric and so difficult to quantify pH. (A) An image of HEK293 cells expressing the sensor in one island of a MOSAIC array. (B) Low magnification image of a confluent layer of HEK293 cells lentivirally transfected with the sensor. (C) An image of hiPS derived cardiomyocytes patterned into two adjacent islands of a MOSAIC array. (D) The pH response of the sensor across the physiological range. Images were recorded every 2 minutes with illumination at 470 nm and emission in the GFP fluorescence channel. To equilibrate the pH with the buffer pH, we add the K^+^/H^+^ exchanger nigericin at 14 μM. To prevent a [K^+^] gradient from driving a proton gradient, we use a high-potassium buffer^22,23^. The buffer composition was (in mM): Good’s zwitterionic buffer 25, KCl 100, NaCl 38, CaCl_2_ 1.8, MgSO_4_ 0.8, NaH_2_PO_4_ 0.9. The Good buffer, chosen for its pK_a_ and effective buffering pH range, was MES for pH 6.0 and 6.5 and HEPES for pH 7 – 8.5. Data is from *n* = 1 dish.


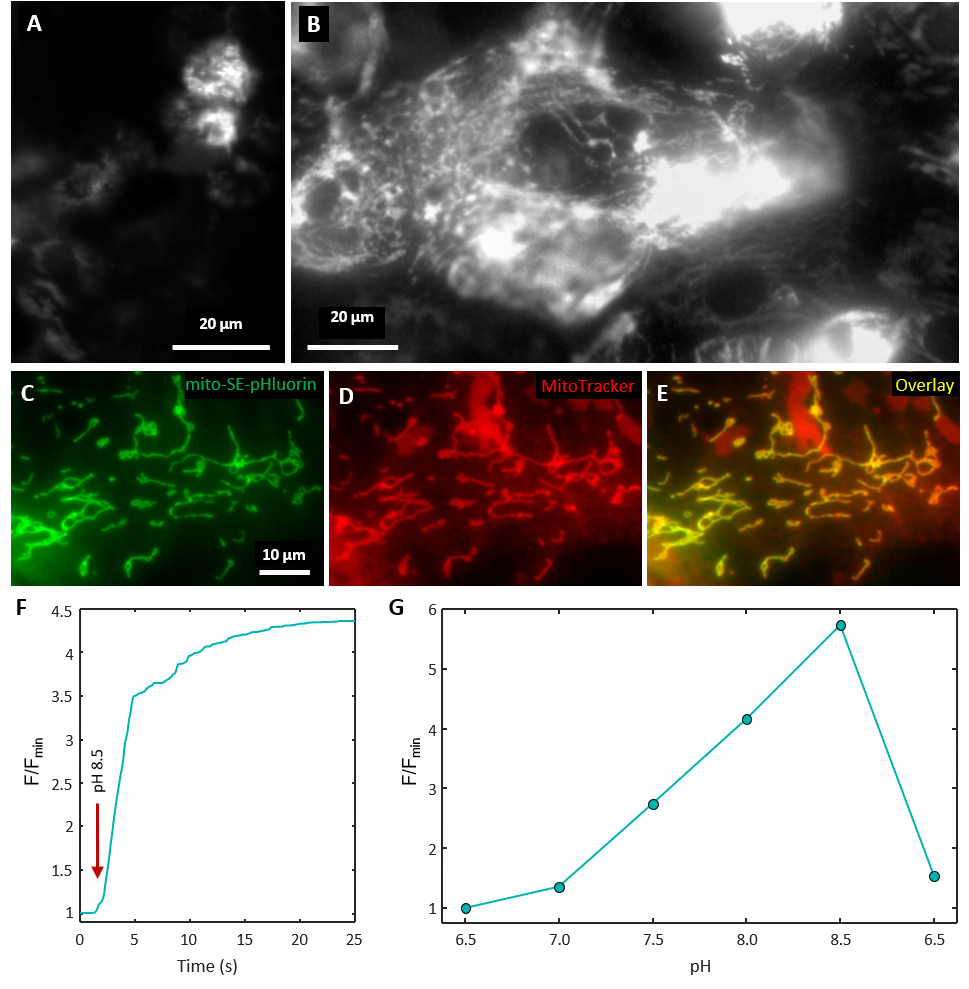


**Supplementary Figure 9 – Mitochondrially-targeted superecliptic pHluorin.** Superecliptic pHluorin, an engineered derivative of EGFP, becomes dimmer at acidic pH due to protonation of the chromophore^22^. Its absorption and emission bands are aligned to those of EGFP. The sensor is bright and highly sensitive, although it is not ratiometric and so difficult to quantify absolute pH. It is targeted to the mitochondria using an N-terminal, in-frame fusion of the 28 amino acid cytochrome oxidase subunit 8 (COX8) pre-sequence^24^. (A) HEK293 cells expressing the sensor. (B) Human iPS-derived cardiomyocytes (CMs) in the MOSAIC array. (C) Long, tubular mitochondria in the CM periphery expressing mito-SE-pHluorin, (D) the same cell with MitoTracker Red FM (ThermoFisher #M22425), a positively charged dye that localizes to the negative resting potential mitochondria, and (E) their overlay. For functional recordings, the sensor was excited at 470 nm and fluorescence was collected in the GFP channel. (F) The temporal response in CMs to an acute pH change from pH 6.5 to 8.5, recorded at 10 Hz showing fast sensor dynamics. (G) The pH response of the sensor across the physiological range. Images were recorded every 2 minutes. To equilibrate the pH with the buffer pH, we add the K^+^/H^+^ exchanger nigericin at 14 μM. To prevent a [K^+^] gradient from driving a proton gradient, we use a high-potassium buffer^22,23^. The buffer composition was (in mM): Good’s zwitterionic buffer 25, KCl 100, NaCl 38, CaCl_2_ 1.8, MgSO_4_ 0.8, NaH_2_PO_4_ 0.9. The Good buffer, chosen for its pK_a_ and effective buffering pH range, was MES for pH 6.0 and 6.5 and HEPES for pH 7 – 8.5. Data is from *n* = 1 dish.


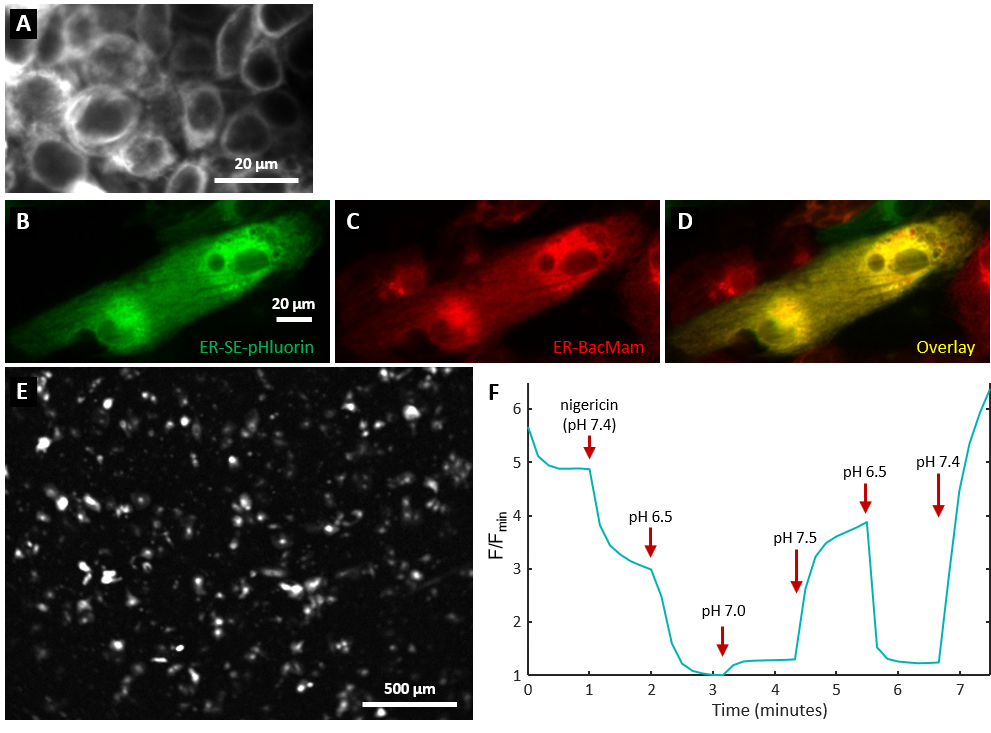


**Supplementary Figure 10 – ER-superecliptic pHluorin.** Superecliptic pHluorin, an engineered derivative of EGFP, becomes dimmer at acidic pH due to protonation of the chromophore^22^. Its absorption and emission bands are aligned to those of EGFP. The sensor is bright and highly sensitive, although it is not ratiometric and so difficult to quantify pH. It is targeted to the endoplasmic reticulum using the MLLSVPLLLGLLGLAAA, N-terminal ER localization sequence from calreticulin and the KDEL C-terminal ER retention sequence^18^. (A) HEK293 cells expressing ER-SE-pHluorin. (B) Fluorescence in a human iPS-derived cardiomyocyte (CM) from ER-SE-pHluorin, (C) the same cell with BacMam ER-RFP (ThermoFisher #C10591), a well-validated construct that traffics to the ER, and (D) their overlay. (E) A widefield view of ER-SE-pHluorin in CMs excited at 485 nm and (F) the average time trace during buffer exchange. The fluorescence tracks with pH across the physiological range. Pushing beyond this range typically induced CMs to peel off the dish. Images were recorded every 10 seconds under 485 nm illumination and recording in the GFP fluorescence channel. To equilibrate the pH of all cellular compartments with the buffer pH, we add the K^+^/H^+^ exchanger nigericin at 14 μM. To prevent a [K^+^] gradient from driving a proton gradient, we use a high-potassium buffer^22,23^. The buffer composition was (in mM): Good’s zwitterionic buffer 25, KCl 100, NaCl 38, CaCl_2_ 1.8, MgSO_4_ 0.8, NaH_2_PO_4_ 0.9. The Good buffer, chosen for its pKa and effective buffering pH range, was MES for pH 6.0 and 6.5 and HEPES for pH 7 – 8.5. The initial imaging buffer did not have nigericin. To analyze, any cells that washed away were masked and not included in analysis. The darkest 70% of pixels were counted as background, and their time trace was subtracted from the average cell trace.


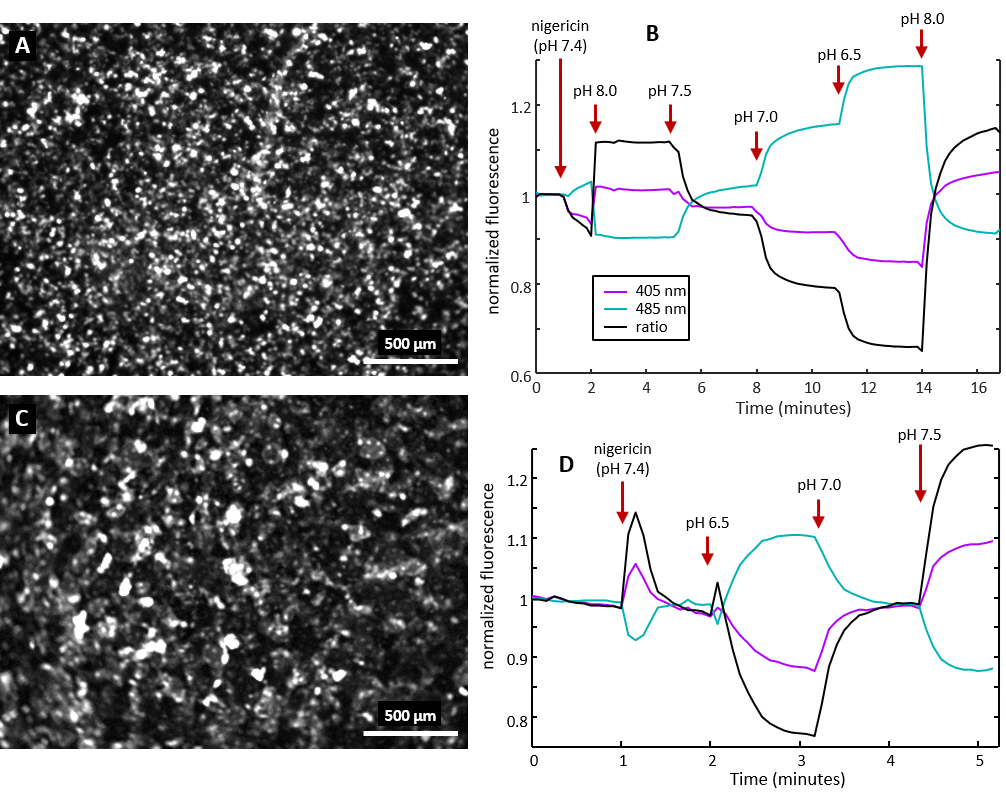


**Supplementary Figure 11 – Cytosolic ratiometric pHluorin.** Ratiometric pHluorin, an engineered derivative of GFP, has absorption bands 405 and 485 nm. Protonation of the chromophore at acidic pH increases fluorescence from the 485 nm absorption band while simultaneously decreasing fluorescence from the 405 nm absorption band^22^. Taking the ratio of fluorescence at the two excitation wavelengths, one can quantify intracellular pH levels^22^. (A) HEK293 cells expressing cyto-ratio-pHluorin excited at 485 nm. (B) Fluorescent time traces while different pH buffers are flowed across the cells using perfusion. (C) Human iPS cell-derived cardiomyocytes (CMs) expressing cyto-ratio-pHluorin excited at 485 nm. (D) The average time trace during buffer exchange. The fluorescence ratio tracks with pH across the physiological range. Pushing beyond this range typically induced CMs to peel off the dish. Images were recorded every 10 seconds under both 405 nm and 485 nm illumination and recording in the GFP fluorescence channel. To equilibrate the pH of all cellular compartments with the buffer pH, we add the K^+^/H^+^ exchanger nigericin at 14 μM. To prevent a [K^+^] gradient from driving a proton gradient, we use a high-potassium buffer^22,23^. The buffer composition was (in mM): Good’s zwitterionic buffer 25, KCl 100, NaCl 38, CaCl_2_ 1.8, MgSO_4_ 0.8, NaH_2_PO_4_ 0.9. The Good buffer, chosen for its pKa and effective buffering pH range, was MES for pH 6.0 and 6.5 and HEPES for pH 7 – 8.5. The initial imaging buffer did not have nigericin. To analyze, any cells that washed away were masked and not included in analysis. For HEK293 cells (CMs), the darkest 15% (75%) pixels were counted as background, and their time trace was subtracted from the average cell trace.


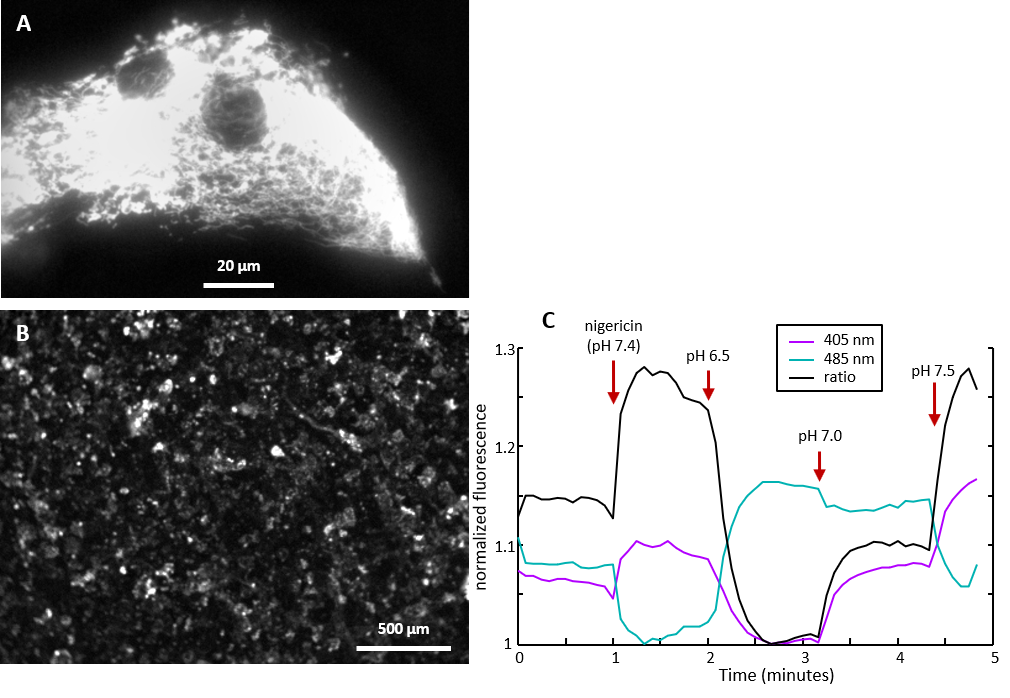


**Supplementary Figure 12 – Mitochondrially-targeted ratiometric pHluorin.** Ratiometric pHluorin, an engineered derivative of GFP, has absorption bands 405 and 485 nm. Protonation of the chromophore at acidic pH increases fluorescence from the 485 nm absorption band while simultaneously decreasing fluorescence from the 405 nm absorption band^22^. Taking the ratio of fluorescence at the two excitation wavelengths, one can quantify intracellular pH levels^22^. Ratiometric pHluorin is targeted to the mitochondria using an N-terminal, in-frame fusion of the 28 amino acid cytochrome oxidase subunit 8 (COX8) pre-sequence^24^. (A) A human iPS-derived cardiomyocyte (CM) showing mitochondrial trafficking. (B) A wide-field view of CMs expressing mito-ratio-pHluorin excited at 485 nm. (C) The average time trace during buffer exchange. The fluorescence ratio tracks with pH across the physiological range. Pushing beyond this range typically induced CMs to peel off the dish. Images were recorded every 10 seconds under both 405 nm and 485 nm illumination and recording in the GFP fluorescence channel. To equilibrate the pH of all cellular compartments with the buffer pH, we add the K^+^/H^+^ exchanger nigericin at 14 μM. To prevent a [K^+^] gradient from driving a proton gradient, we use a high-potassium buffer^22,23^. The buffer composition was (in mM): Good’s zwitterionic buffer 25, KCl 100, NaCl 38, CaCl_2_ 1.8, MgSO_4_ 0.8, NaH_2_PO_4_ 0.9. The Good buffer, chosen for its pKa and effective buffering pH range, was MES for pH 6.0 and 6.5 and HEPES for pH 7 – 8.5. The initial imaging buffer did not have nigericin. To analyze, any cells that washed away were masked and not included in analysis. The darkest 35% of pixels were counted as background, and their time trace was subtracted from the average cell trace.


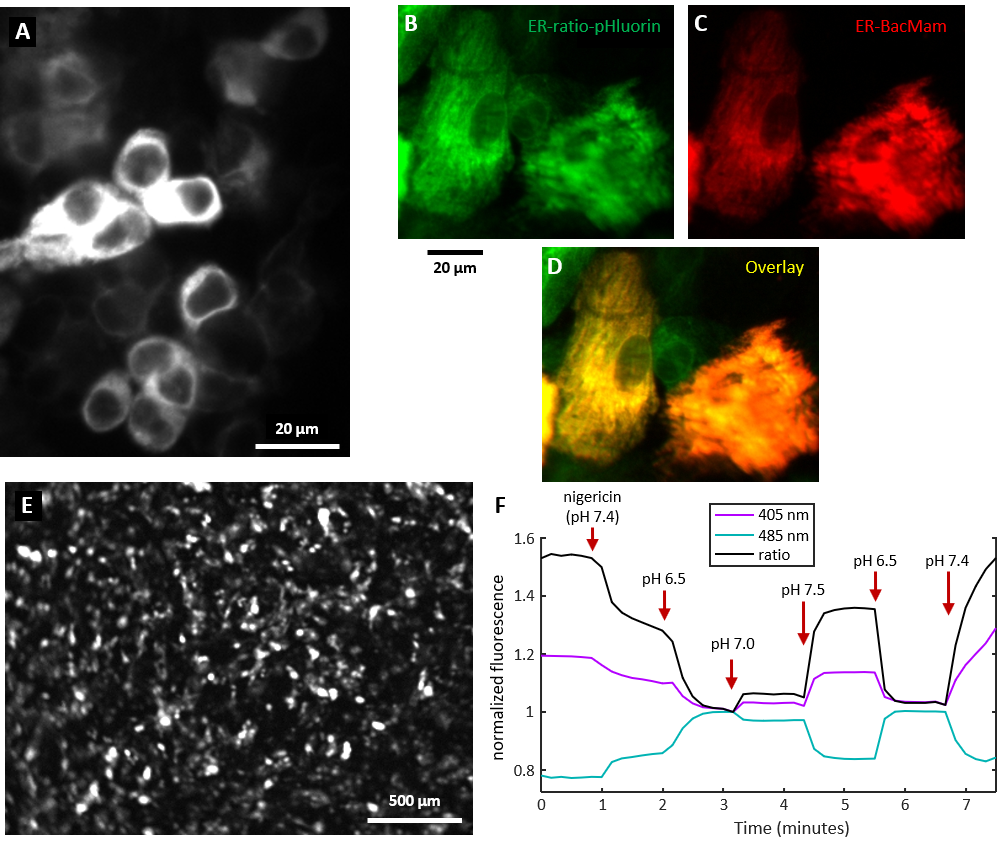


**Supplementary Figure 13 – ER-ratiometric pHluorin.** Ratiometric pHluorin, an engineered derivative of GFP, has absorption bands 405 and 485 nm. Protonation of the chromophore at acidic pH increases fluorescence from the 485 nm absorption band while simultaneously decreasing fluorescence from the 405 nm absorption band^22^. Taking the ratio of fluorescence at the two excitation wavelengths, one can quantify intracellular pH levels^22^. Ratiometric pHluorin is targeted to the endoplasmic reticulum using the MLLSVPLLLGLLGLAAA, N-terminal ER localization sequence from calreticulin and the KDEL C-terminal ER retention sequence^18^. (A) HEK293 cells expressing ER-ratio-pHluorin excited at 488 nm and collected in the GFP fluorescence channel. (B) Fluorescence in a human iPS-derived cardiomyocyte (CM) from ER-SE-pHluorin, (C) the same cell with BacMam ER-RFP (ThermoFisher #C10591), an ER marker, and (D) their overlay. (E) A widefield view of ER-SE-pHluorin in CMs excited at 485 nm and (F) the average time trace during buffer exchange. The fluorescence tracks with pH across the physiological range. Pushing beyond this range typically induced CMs to peel off the dish. Images were recorded every 10 seconds under 485 nm illumination and recording in the GFP fluorescence channel. To equilibrate the pH of all cellular compartments with the buffer pH, we add the K^+^/H^+^ exchanger nigericin at 14 μM. To prevent a [K^+^] gradient from driving a proton gradient, we use a high-potassium buffer^22,23^. The buffer composition was (in mM): Good’s zwitterionic buffer 25, KCl 100, NaCl 38, CaCl_2_ 1.8, MgSO_4_ 0.8, NaH_2_PO_4_ 0.9. The Good buffer, chosen for its pKa and effective buffering pH range, was MES for pH 6.0 and 6.5 and HEPES for pH 7 – 8.5. The initial imaging buffer did not have nigericin. To analyze, any cells that washed away were masked and not included in analysis. The darkest 70% of pixels were counted as background, and their time trace was subtracted from the average cell trace.


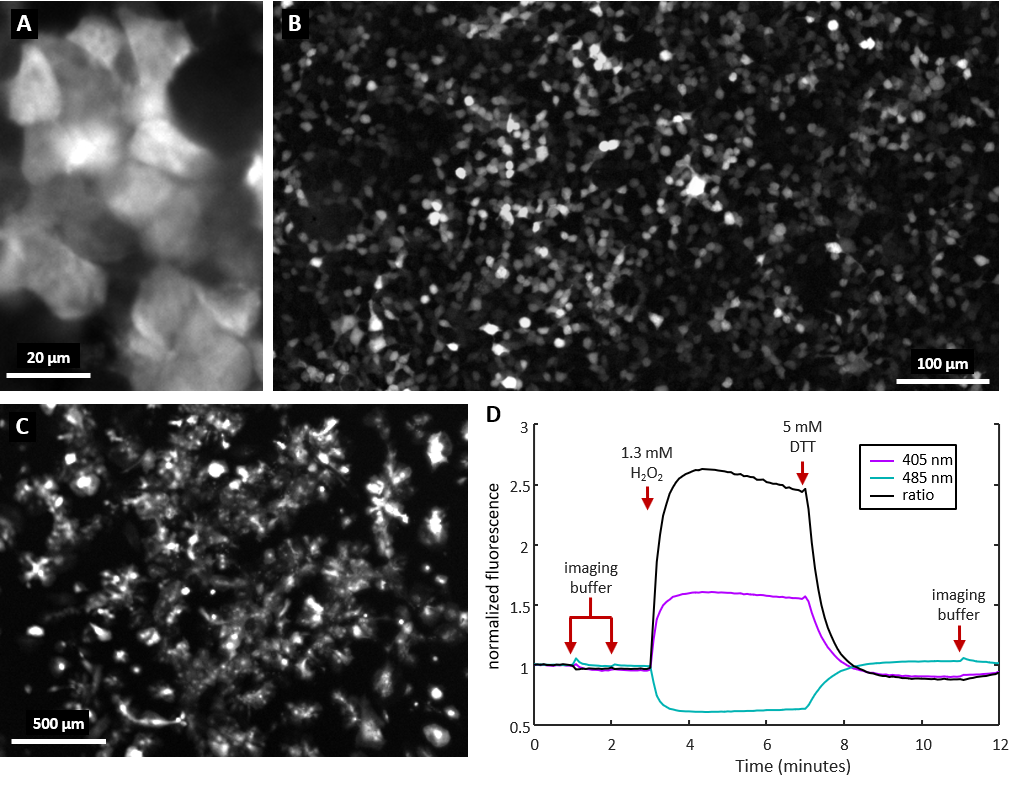


**Supplementary Figure 14 – Cytosolic Grx1-roGFP2.** This reporter is based around the redox-sensitive roGFP2, which was designed by adding a strained disulfide bond into EGFP^25,26^. The sensor is fused to *Grx*1, Glutaredoxin-1, which rapidly equilibrates the roGFP2 redox potential with the cellular glutathione redox potential by catalyzing the making and breaking of disulfide bonds^27–29^. (A) A magnified image of the sensor in HEK293 cells showing relatively homogeneous expression throughout the cytosol. (B) A low magnification view showing the lentiviral transfection efficiency. (C) A low magnification image of cardiomyocytes lentivirally transfected with the sensor and (D) their response to changes in cellular redox potential. Fluorescence images were collected every or 5 sec in the GFP channel; the sensor was excited with 405 nm and 485 nm light, and the readout is calculated from the ratio *F*_405_*/F*_485_. Cells were oxidized with hydrogen peroxide and reduced with dithiothreitol (DTT).


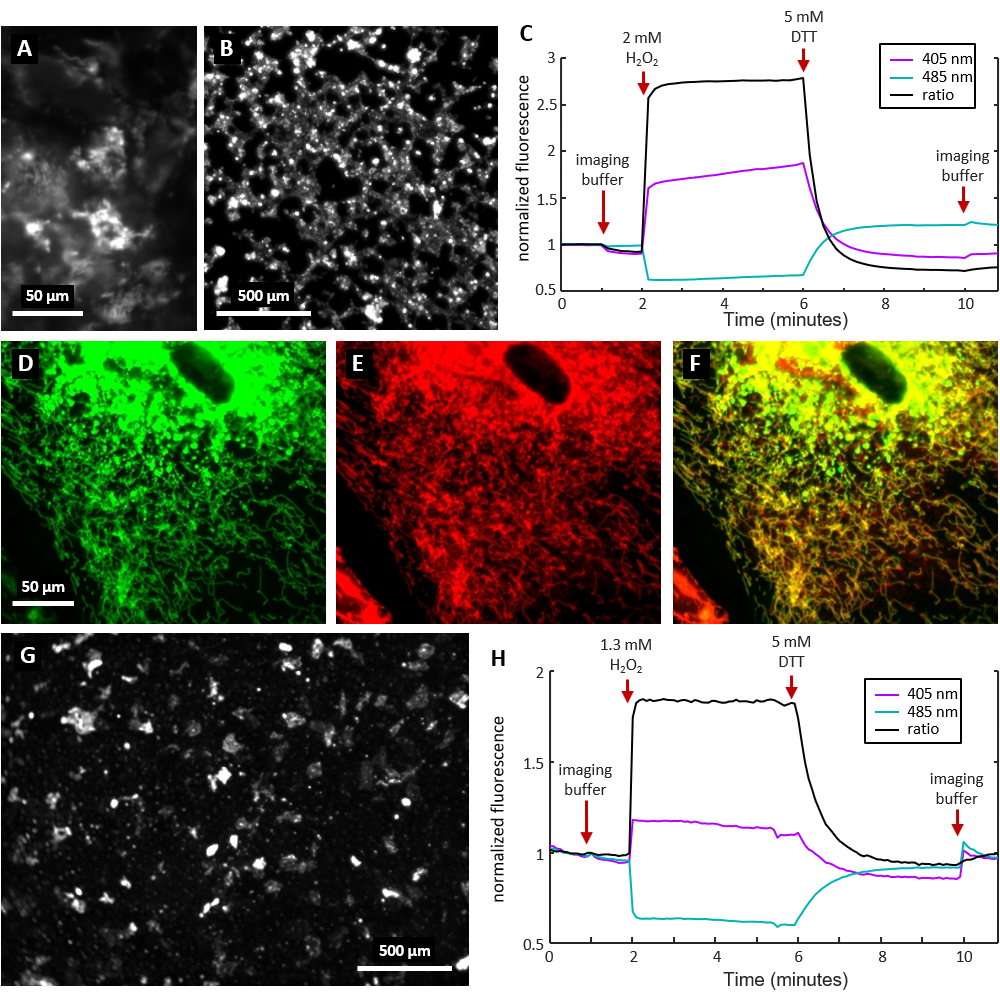


**Supplementary Figure 15 – Mitochondrially-targeted roGFP2-Grx1.** This reporter is based around the redox-sensitive roGFP2, which was designed by adding a strained disulfide bond into EGFP^25,26^. The sensor is fused to *Grx*1, Glutaredoxin-1, which rapidly equilibrates the roGFP2 redox potential with the cellular glutathione redox potential by catalyzing the making and breaking of disulfide bonds^27–29^. roGFP2-Grx1 is targeted to the mitochondria matrix with the signal sequence from *Neurospora crassa* ATP synthase protein 9^27^. Fluorescence images are collected every 10 sec (HEK293) or 5 sec (cardiomyocytes) in the GFP channel, the sensor is excited with 405 nm & 485 nm light, and the readout is calculated from the ratio *F*_405_*/F*_485_. Cells are fully oxidized with hydrogen peroxide, and fully reduced with dithiothreitol (DTT). (A)-(C) HEK293 cells. (D)-(H) Cardiomyocytes. (A) A magnified image showing trafficking. (B) A low magnification view and (C) their fluorescence time trace. The mitochondria begin predominantly reduced, oxidize rapidly upon hydrogen peroxide addition, and become slightly more reduced than baseline in response to DTT. (D) A high-magnification image of the sensor showing long, tubular mitochondria in the cell periphery, (E) the same cell with MitoTracker Red FM (ThermoFisher #M22425), a positively charged dye that localizes to the negative resting potential mitochondria, and (F) their overlay. (G) A low magnification image of cardiomyocytes expressing the sensor and (H) their response to changes in cellular redox potential.


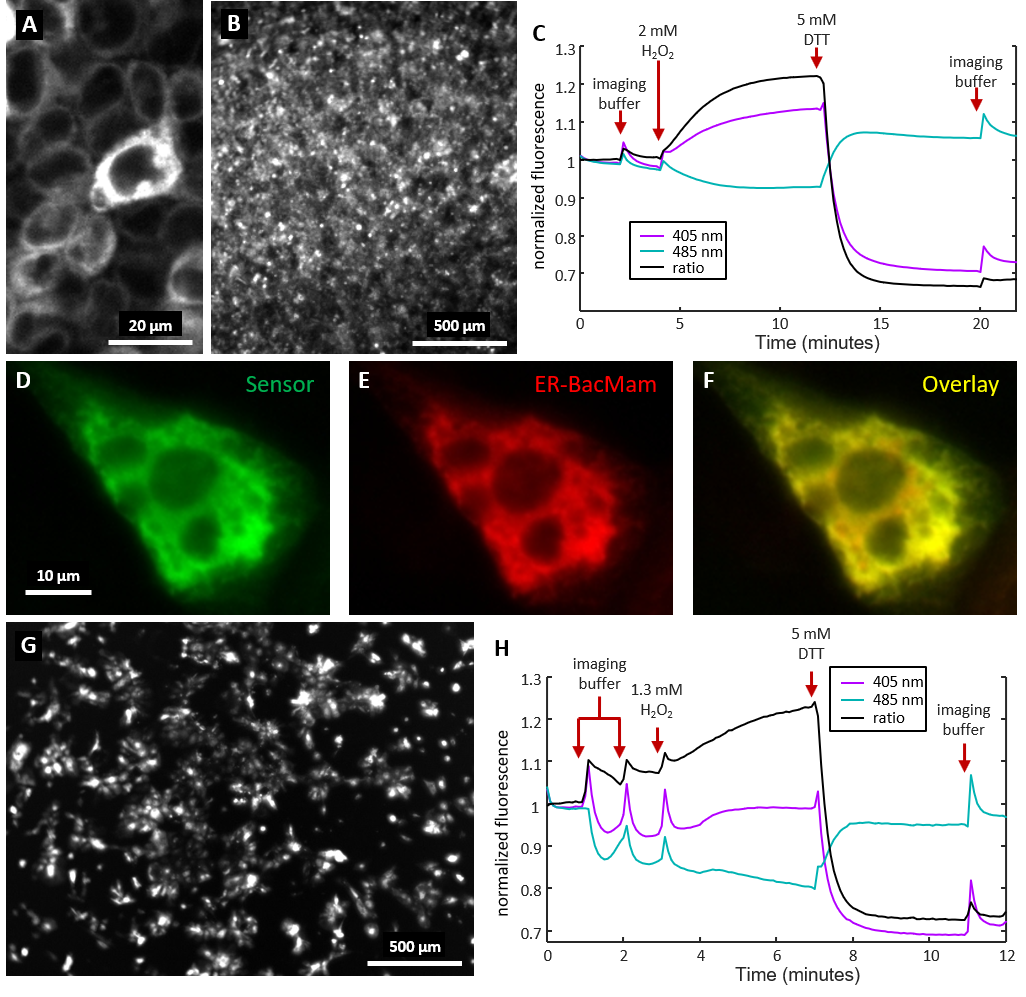


**Supplementary Figure 16 – Endoplasmic reticulum-targeted Grx1-roGFP1-iE_ER_.** This reporter is based around the redox-sensitive roGFP1, which was designed by adding a strained disulfide bond into GFP^25,26^. In contrast to the other redox sensors used in the study, which are optimized for the reducing environment in the cytosol or mitochondria, this redox sensor is tuned to report on redox changes to the oxidizing environment of the ER using roGFP1-iE^30^. The sensor is fused to *Grx*1, Glutaredoxin-1, which rapidly equilibrates the roGFP2 redox potential with the cellular glutathione redox potential by catalyzing the making and breaking of disulfide bonds^27^. Grx1-roGFP1-iE is targeted to the ER with an N-terminal ER signaling peptide and C-terminal ER retrieval motif [3]. Fluorescence images are collected every 10 sec. (HEK293) or 5 sec. (cardiomyocytes) in the GFP channel, the sensor is excited with 405 nm & 485 nm light, and the readout is calculated from the ratio *F*_405_*/F*_485_. Cells are fully oxidized with hydrogen peroxide, and fully reduced with dithiothreitol (DTT). (A)-(C) HEK293 cells. (D)-(H) Cardiomyocytes. (A) A magnified image (excited at 488 nm) showing trafficking. (B) A low magnification view (excited at 405 nm) and (C) its fluorescence time trace. The ER begins partially oxidized, oxidizes more upon hydrogen peroxide addition, and becomes highly reduced in response to DTT. (D) A high-magnification image of the sensor in one cardiomyocyte showing ER-localization, (E) the same cell with BacMam ER-RFP (ThermoFisher #C10591), an ER marker, and (F) their overlay. (G) Human iPS-derived cardiomyocytes expressing the sensor and (H) their response to changes in cellular redox potential.


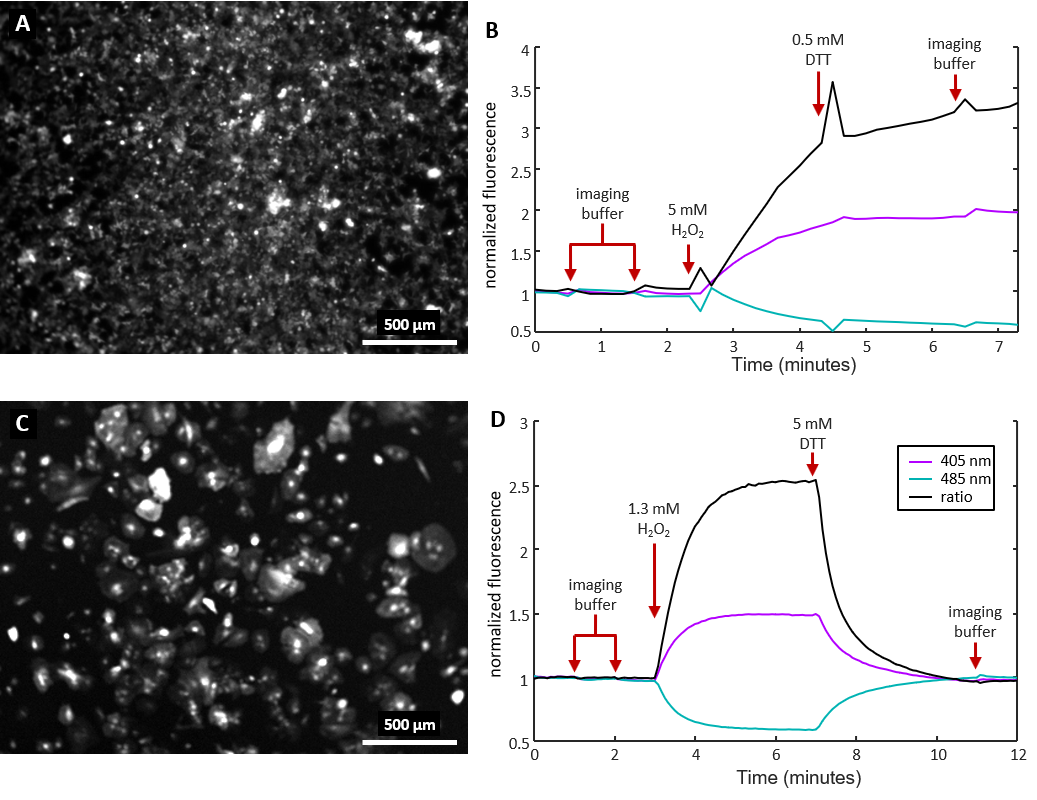


**Supplementary Figure 17 – Cytosolic roGFP2-Orp1.** This reporter is based around the redox-sensitive roGFP2, which was designed by adding a strained disulfide bond into EGFP^25,26^. The sensor is fused to the preoxidase Orp1, which rapidly oxidizes roGFP2 in the presence of hydrogen peroxide; reduction relies on endogenous cellular mechanisms^28,31^. Fluorescence images are collected every 10 sec. (HEK293) and every 5 sec. (cardiomyocytes) in the GFP channel, the sensor is excited with 405 nm & 485 nm light, and the readout is calculated from the ratio *F*_405_*/F*_485_. The background fluorescence is calculated from the average counts in the black regions of the images and subtracted. Cells are fully oxidized with hydrogen peroxide, and fully reduced with dithiothreitol (DTT). A. A widefield image of HEK293 cells expressing this reporter and B. the fluorescence time trace from these cells. The DTT concentration was insufficient to reduce the medium, and so had a minimal effect on cellular redox state. C. A widefield image of cardiomyocytes expressing this reporter and D. the fluorescence time trace from these cells.


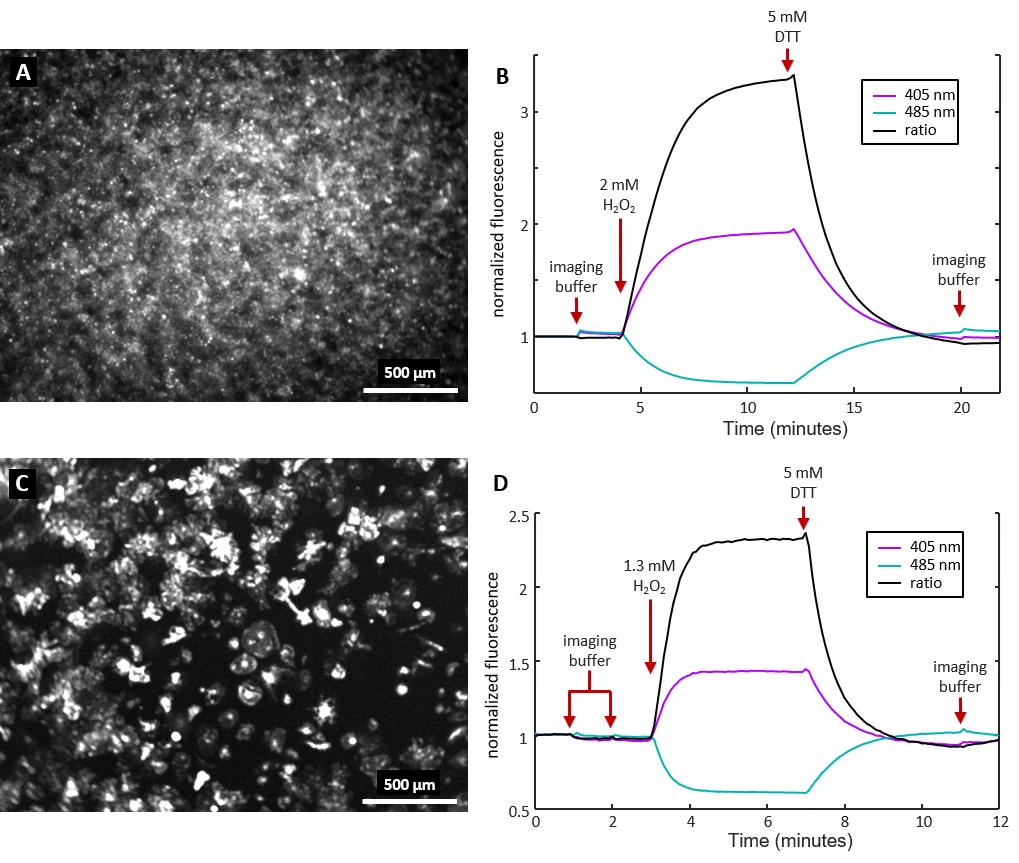


**Supplementary Figure 18 – Mitochondrially-targeted roGFP2-Orp1.** This reporter is based around the redox-sensitive roGFP2, which was designed by adding a strained disulfide bond into EGFP^25,26^. The sensor is fused to the preoxidase Orp1, which rapidly oxidizes roGFP2 in the presence of hydrogen peroxide; reduction relies on endogenous cellular mechanisms^28,31^. roGFP2-Grx1 is targeted to the mitochondria matrix with the signal sequence from *Neurospora crassa* ATP synthase protein 9^27^. Fluorescence images are collected every 10 sec. (HEK293) and every 5 sec. (cardiomyocytes) in the GFP channel, the sensor is excited with 405 nm & 485 nm light, and the readout is calculated from the ratio *F*_405_*/F*_485_. The background fluorescence is calculated from the average counts in the black regions of the images and subtracted. Cells are fully oxidized with hydrogen peroxide, and fully reduced with dithiothreitol (DTT). (A) HEK293 cells expressing this reporter and (B) the fluorescence time trace from these cells. (C) Human, iPSC-derived cardiomyocytes expressing this reporter and D. the fluorescence time trace from these cells.


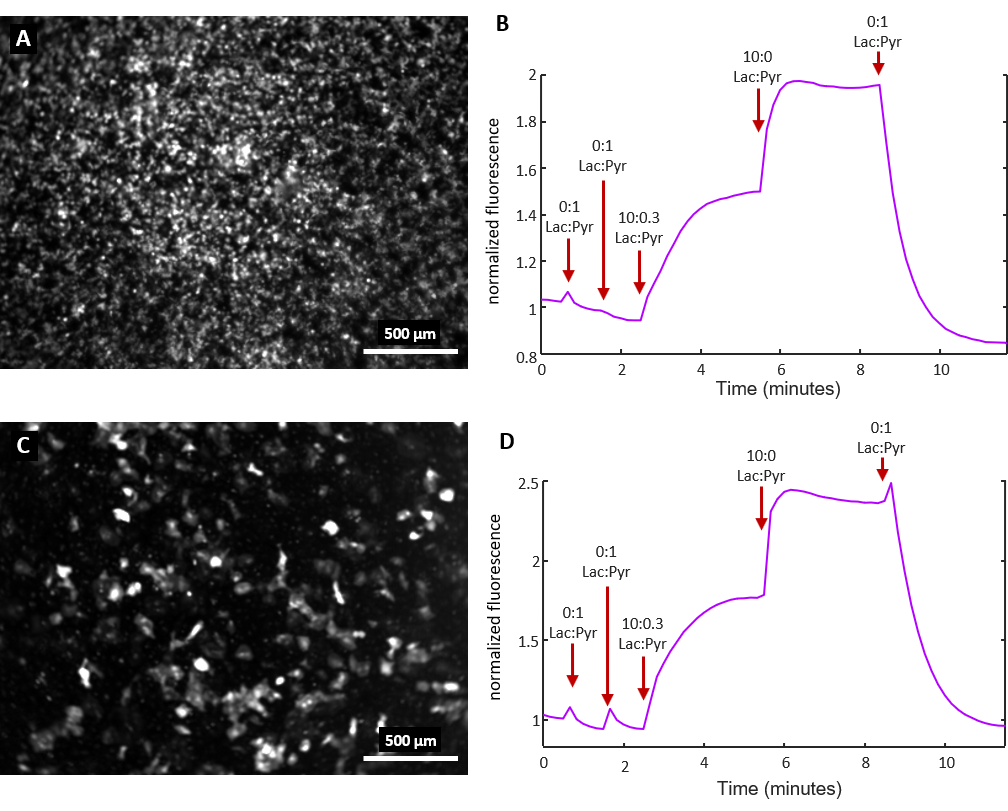


**Supplementary Figure 19 – Peredox NADH:NAD^+^ ratio sensor.** This reporter, based on the circularly permuted Sapphire fluorescent protein, absorbs 405 nm light and emits fluorescence in the GFP fluorescence channel. It reports on the NADH:NAD^+^ ratio^32,33^, giving a readout for cellular respiration and redox status within the cell. Here, we recorded images every 10 seconds, and perfused buffers with different ratios of lactate:pyruvate. The numbers in the figure indicate the lactate and pyruvate concentrations in mM. Lactate and pyruvate enter cells via monocarboxylate transporters, equilibrate readily between extracellular and intracellular compartments, and lead to exchange between NADH and NAD^+^ catalyzed by endogenous lactate dehydrogenases (LDH)^32,33^. (A) Widefield Peredox fluorescence in lentivirally transfected HEK293 cells and (B) their fluorescent time trace. (C) Widefield Peredox fluorescence in lentivirally transfected cardiomyocytes and (D) their fluorescent time trace.


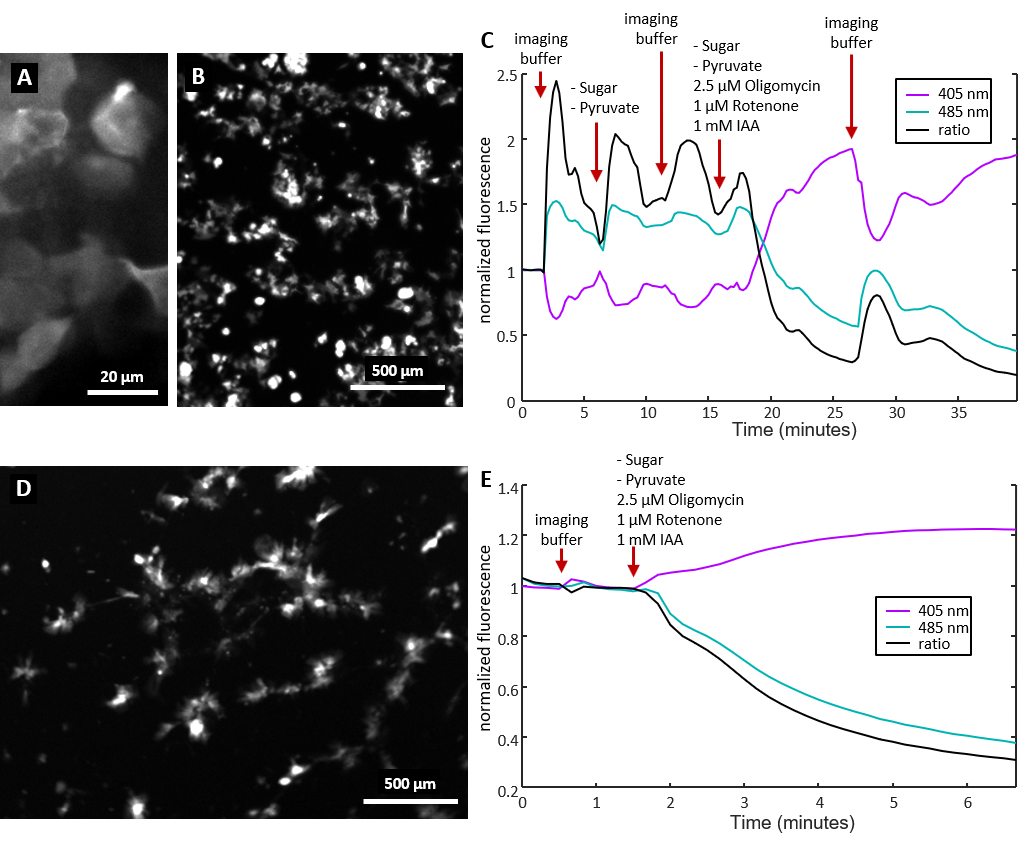


**Supplementary Figure 20 – PercevalHR ATP:ADP ratio sensor.** This reporter is based on a circularly permuted mVenus fluorescent protein fused to ATP-binding bacterial protein GlnK1. It fluoresces in the GFP channel; when excited at 485 nm the fluorescence scales with the ATP:ADP ratio as the two compete for the binding site. When excited at 405 nm, the fluorescence scales inversely, so the *F*_485_/*F*_405_ ratio scales with the ATP:ADP ratio independent of sensor concentration^34,35^. To modulate the ATP:ADP ratio, we exchange the standard imaging buffer (10 mM galactose & 1 mM pyruvate) to a buffer free of energy sources (0 mM sugar, 0 mM pyruvate) with compounds to arrest energy production in the cell: 2.5 μM Oligomycin blocks ATP synthase, 1 μM Rotenone blocks mitochondrial complex I electron transport chain, and 1 mM Iodo acetic acid (IAA) arrests glycolysis by interfering with glyceraldehyde 3-phosphate dehydrogenase. Cardiomyocytes, which have glycogen stores, are facile at maintaining ATP levels without extreme pharmacological intervention. We recorded images every 15 sec (HEK293) or 10 sec (cardiomyocytes) and perfused with the energy deprivation buffer. (A) A high-magnification of HEK293 cells expressing the sensor. (B) Widefield PercevalHR fluorescence in lipofectamine (Mirus TransIT-293) transfected HEK293 and (C) their fluorescent time trace. Rapid ATP (or pH) fluctuations are observed in response to flow, as well as a large drop in ATP levels upon addition of the toxins. (D) Widefield PercevalHR fluorescence in lentivirally transfected cardiomyocytes and (E) their fluorescent time trace.


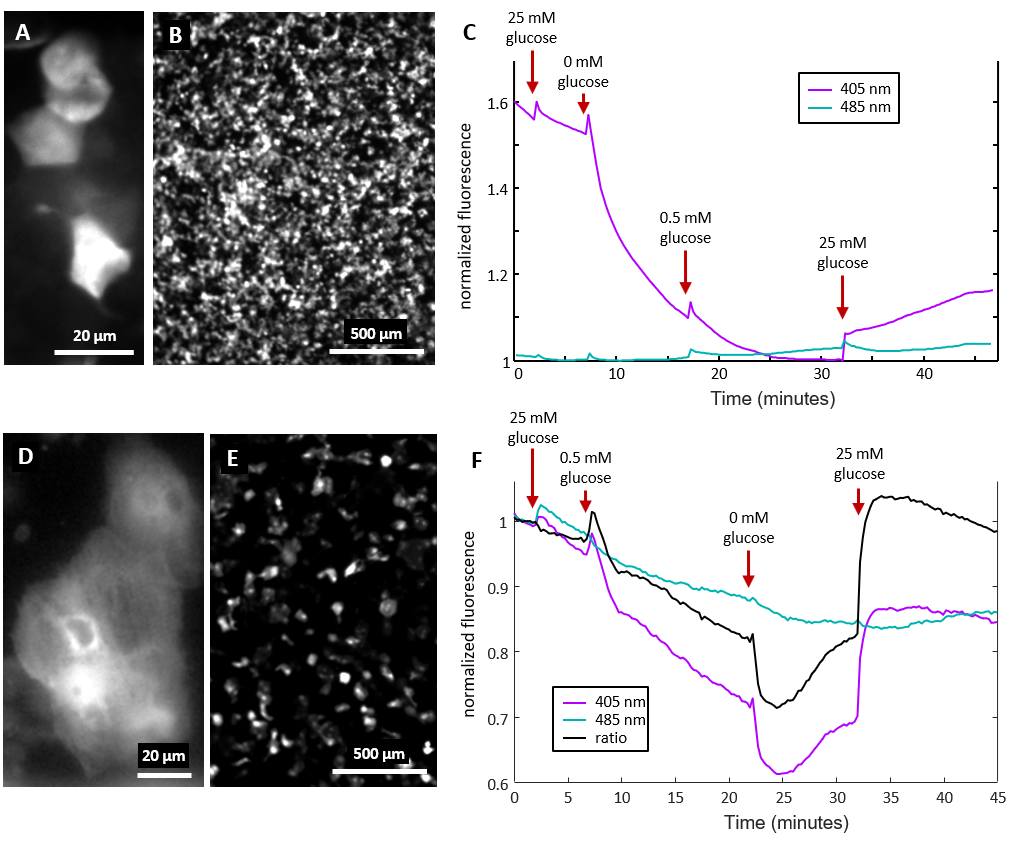


**Supplementary Figure 21 – FLII^12^Pglu-700μδ6 glucose sensor.** This reporter is an ECFP/EYFP FRET-based glucose sensor^36^. Although typically one would stimulate ECFP and record both ECFP and EYFP fluorescence, ECFP fluorescence recording was not compatible with other MOSAIC sensors. To maintain compatibility, we stimulated the ECFP with 405 nm light and record the FRET signal from the EYFP in the GFP fluorescence channel. EYFP fluorescence not mediated through FRET was recorded under 485 nm illumination and imaging in the GFP fluorescence channel; normalization to this trace corrects some fluorescence changes that are not mediated by glucose concentration. Images were recorded every 15 seconds. (A) EYFP fluorescence in lentivirally transduced HEK293 cells stimulated with 488 nm and recorded on a spinning disk confocal microscope. (B) Widefield EYFP fluorescence in HEK293 cells where the ECFP is excited at 405 nm. (C) The EYFP fluorescent time traces from the FOV in (B) as buffers with different glucose concentrations are flowed over the cells. The teal trace, where EYFP is excited directly by 485 nm light, is insensitive to glucose concentration while the 405 nm excited fluorescence operates through FRET and reports on glucose concentration. The fractional fluorescence change is consistent with published values^36^. Interestingly, a close inspection of the movie reveals that different cells get bright at different times after the addition of 25 mM glucose, likely indicating a sudden change in the regulation of glucose transporters across the cell membrane. (D) Sensor fluorescence in cardiomyocytes. Notice subtle banding, likely reflective of muscle fibers. (E) EYFP fluorescence in iPS-CMs excited at 485 nm and (F) the fluorescent time trace. To extract traces in (E) the image was masked over responsive cells.


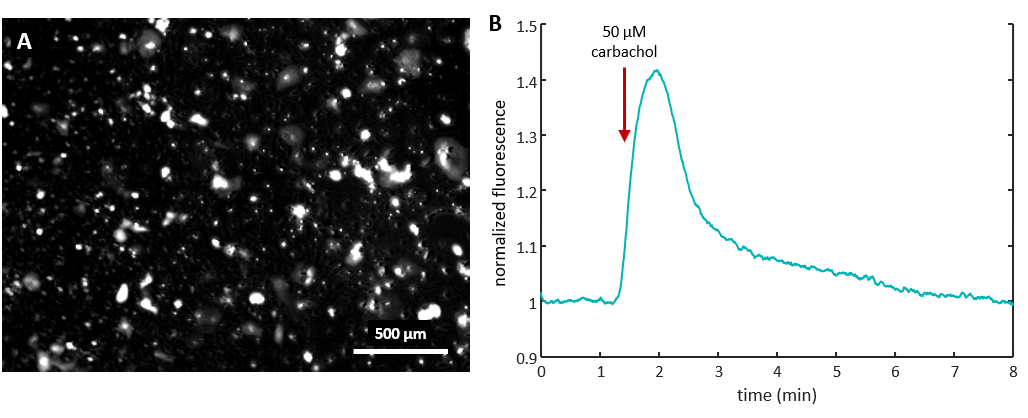


**Supplementary Figure 22 – Upward DAG sensor.** This reporter functions by connecting a circularly permuted GFP (cpGFP) to PKC and the C1 domain. In the absence of secondary messenger diacylglycerol (DAG) the cpGFP β-barrel exposes the chromophore to water, quenching the fluorescence. In the presence of DAG the β-barrel closes and the chromophore becomes bright^37,38^. We excited with 485 nm light and collected fluorescence in the GFP channel at a frame rate of 2 Hz. (A) A widefield fluorescence image of iPS-derived cardiomyocytes expressing Upward DAG; the large dimmer cells are responsive to acetylcholine signaling. (B) The fluorescence time trace of those cells in response to 50 μM carbachol, an acetylcholine analog that that stimulates both muscarinic and nicotinic acetylcholine receptors, triggering the *Gα_q_* signaling pathway and subsequent cleavage of PIP_2_ into iP_3_ and DAG by the enzyme PLC^38^. Acetylcholine signaling is critical in cardiomyocytes to regulate beat rate, and iPS cardiomyocyte beating slows in response to carbachol.


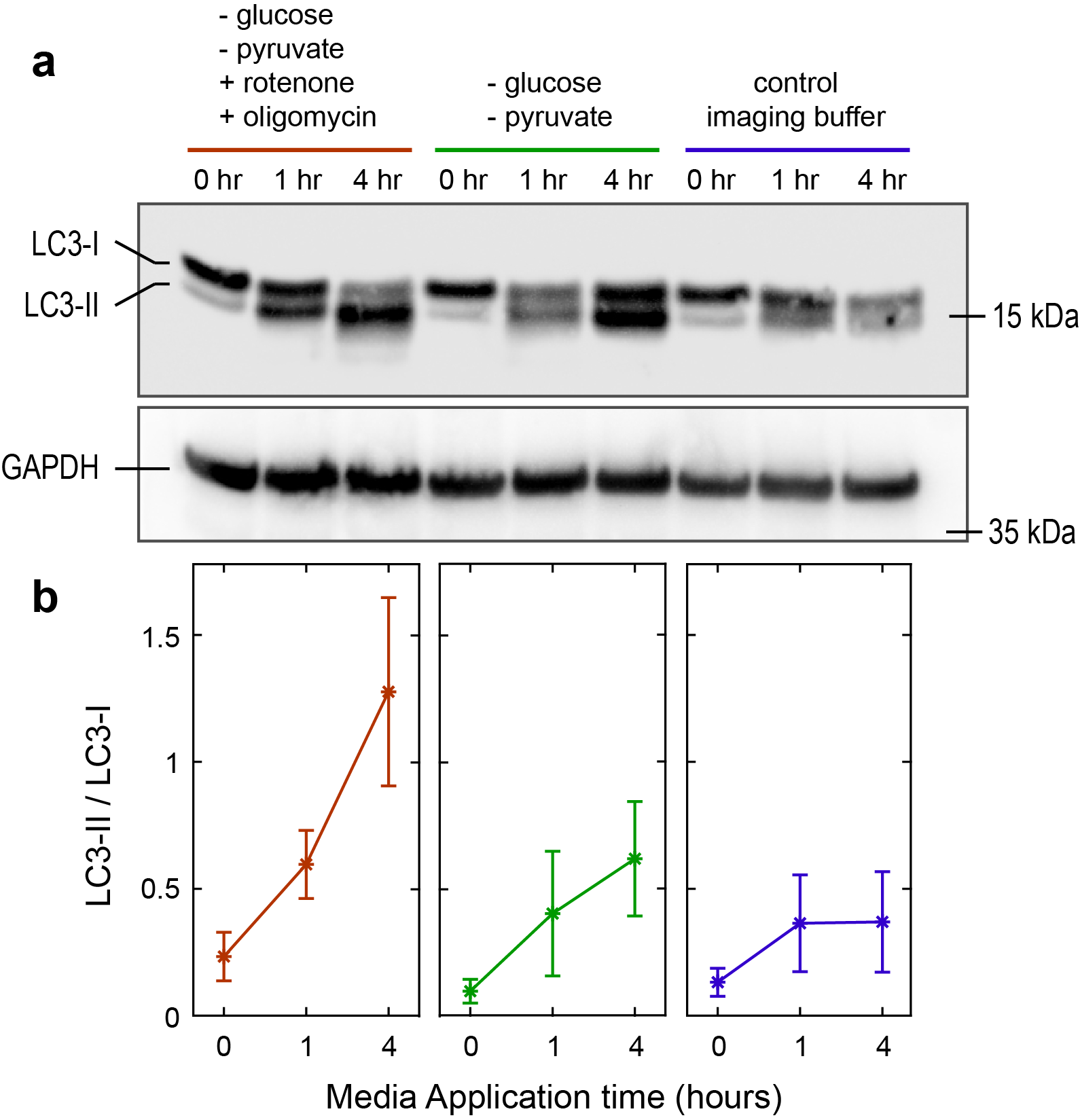


**Supplementary Figure 23 – Energy deprivation-induced autophagic flux.** HEK293 cells were treated for two hours with 100 nM Bafilomycin A_1_, which blocks V-ATPase and inhibits lysosome acidification, thus slowing autophagosome degradation by autophagosome/lysosome fusion^39^. The rate of autophagosome accumulation, reported here by the ratio of LC3-II to LC3-I, is a measure of overall autophagic activity. Upon activation of autophagy, the cytosolic protein LC3-I is lipidated to form LC3-II, which is trafficked to and maintained on the inner phagophore membrane surface. After Bafilomycin treatment, media was exchanged to 1. standard imaging buffer with glucose and pyruvate (blue), 2. imaging buffer without glucose or pyruvate (green), or 3. imaging buffer without glucose or pyruvate and pharmacological inhibitors of cellular respiration. Here we omitted glycolysis blocker IAA, which was applied in combination with rotenone and oligomycin for 10 minutes in Fig. 5, because the HEK293 cells could not survive for 4 hours with complete inhibition of energy production. **a** A western blot showing LC3-I, LC3-II, and housekeeping protein GAPDH at three timepoints after each imaging buffer treatment. The samples LC3 and GAPDH samples derive from the same experiment and the gels were processed in parallel. **b** The ratio of band intensity, LC3-II / LC3-I, averaged over *n = 3* independent rounds of cell plating and measurement. Error bars are standard error of the mean (SEM). Full energy deprivation (red) shows an increase in autophagic flux; the increase from removal of glucose and pyruvate alone is smaller. Source data are provided as a **Source Data file**.


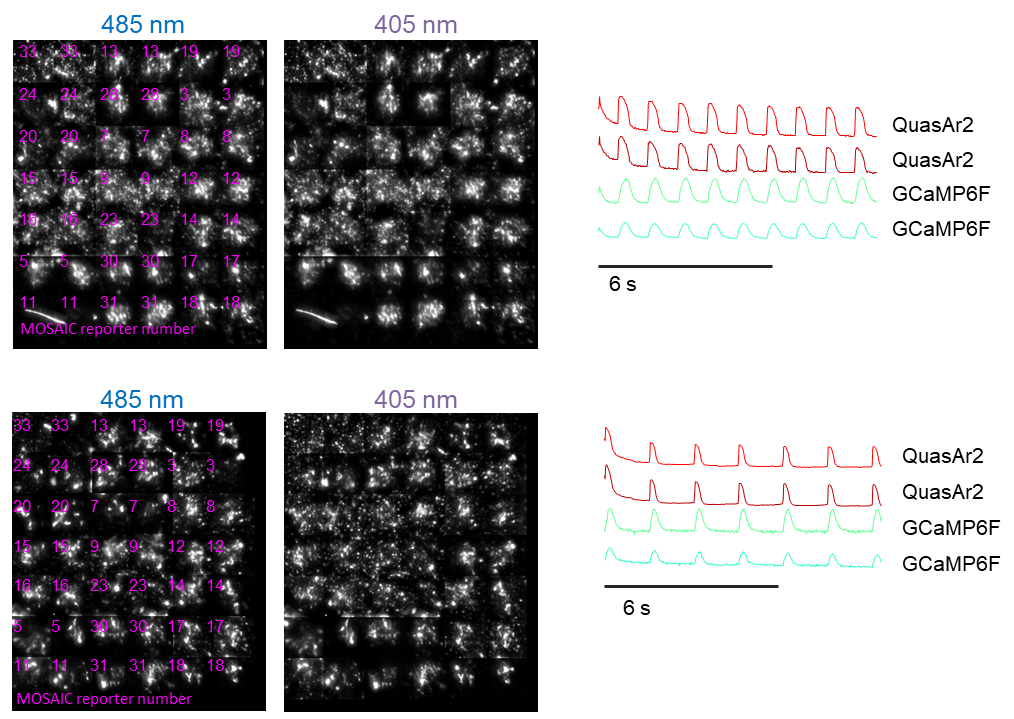


**Supplementary Figure 24 – Multiple MOSAIC arrays made with hiPSC-CM.** Images show MOSAIC arrays imaged with fluorescence at 485 nm (left) and 405 nm (right). In these images the contrast has been individually adjusted for each MOSAIC island to account for island-to-island variation in absolute brightness.

**
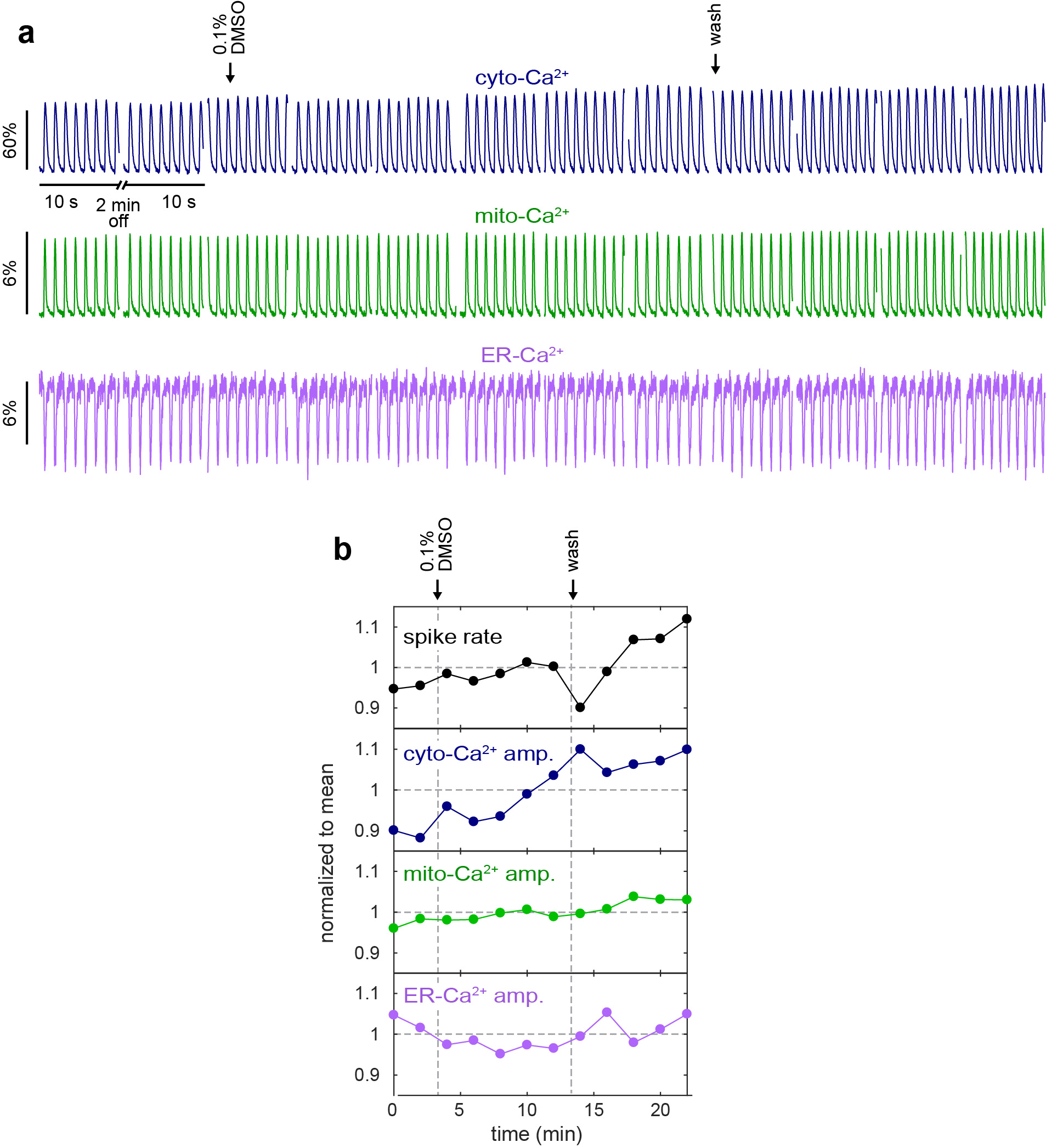
**

**Supplementary Figure 25 – hiPSC-CM washing control data.** MOSAIC recordings from calcium sensors GCaMP6F (cytosolic calcium), mitycam (mitochondrial calcium), and GCaMPer (ER calcium) in spontaneously beating hiPSC-CMs. Cardiomyocytes show modest changes in behavior in response to washes with control imaging buffer, with or without 1% DMSO. Recordings are from a MOSAIC array matching the layout of that in Fig. 7, except with the voltage sensor QuasAr replaced by CheRiff. To stabilize initial conditions, cells have been incubated on the microscope for 20 minutes and repeatedly perfused prior to the recording. **a** Fluorescence recordings (10 sec @ 50 Hz frame rate every two minutes). **b** Average beat rate and calcium transient amplitudes in each epoch, each normalized to the mean. They vary by $\lesssim\pm10\%$.


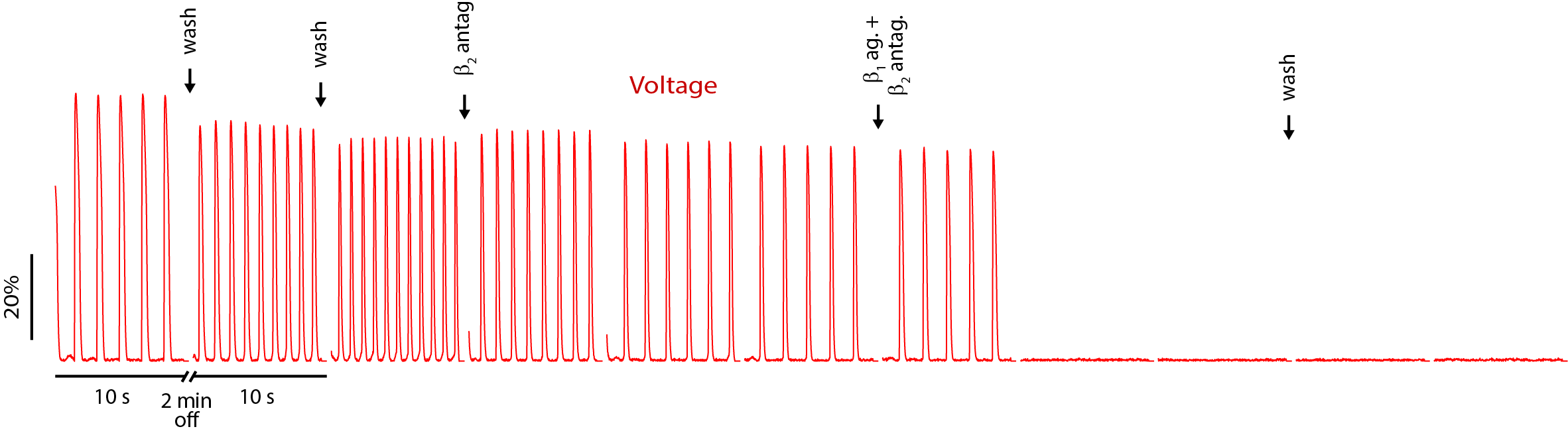


**Supplementary Figure 26 – hiPSC-CM response to dobutamine.** Voltage recordings in CDI human cardiomyocytes using the QuasAr voltage sensor. β_2_ antagonist: ICI-118,551, 100 nM. β_1_ agonist: Dobutamine, 100 μM. Although Dobutamine would be expected to increase heart rate as a β_1_-adrenergic agonist, at these doses in these cells it leads to a cessation of beating.

**Supplementary References**

1. Werley, C. A., Chien, M.-P. & Cohen, A. E. An ultrawidefield microscope for high-speed fluorescence imaging and targeted optogenetic stimulation. *Biomed. Opt. Express* **8**, 5794 (2017).

2. Hochbaum, D. R. *et al.* All-optical electrophysiology in mammalian neurons using engineered microbial rhodopsins. *Nat. Methods* **11**, 825–833 (2014).

3. Werley, C. A. *et al.* All-optical electrophysiology for disease modeling and pharmacological characterization of neurons. *Curr. Protoc. Pharmacol.* **2017**, 11.20.1-11.20.24 (2017).

4. Lou, S. *et al.* Genetically Targeted All-Optical Electrophysiology with a Transgenic Cre-Dependent Optopatch Mouse. **36**, 11059–11073 (2016).

5. Dempsey, G. T. *et al.* Cardiotoxicity screening with simultaneous optogenetic pacing, voltage imaging and calcium imaging. *J. Pharmacol. Toxicol. Methods* **81**, 240–250 (2016).

6. Werley, C. A. *et al.* Geometry-dependent functional changes in iPSC-derived cardiomyocytes probed by functional imaging and RNA sequencing. *PLoS One* **12**, e0172671 (2017).

7. Zhang, H., Reichert, E. & Cohen, A. E. Optical electrophysiology for probing function and pharmacology of voltagegated ion channels. *Elife* **5**, e15202 (2016).

8. McNamara, H. M., Zhang, H., Werley, C. A. & Cohen, A. E. Optically controlled oscillators in an engineered bioelectric tissue. *Phys. Rev. X* **6**, 031001 (2016).

9. Williams, L. A. *et al.* Scalable Measurements of Intrinsic Excitability in Human iPS Cell-Derived Excitatory Neurons Using All-Optical Electrophysiology. *Neurochem. Res.* **44**, 714–725 (2019).

10. Akerboom, J. *et al.* Optimization of a GCaMP Calcium Indicator for Neural Activity Imaging. *J. Neurosci.* **32**, 13819–13840 (2012).

11. Chen, T.-W. *et al.* Ultrasensitive fluorescent proteins for imaging neuronal activity. *Nature* **499**, 295–300 (2013).

12. Touyz, R. M. *et al.* Cytosolic Calcium Changes Induced by Angiotensin II in Neonatal Rat Atrial and Ventricular Cardiomyocytes Are Mediated via Angiotensin II Subtype 1 Receptors. *Hypertension* **27**, 1090–1096 (1996).

13. Terhzaz, S. *et al.* Differential gel electrophoresis and transgenic mitochondrial calcium reporters demonstrate spatiotemporal filtering in calcium control of mitochondria. *J. Biol. Chem.* **281**, 18849–18858 (2006).

14. Kettlewell, S. *et al.* Changes of intra-mitochondrial Ca^2+^ in adult ventricular cardiomyocytes examined using a novel fluorescent Ca^2+^ indicator targeted to mitochondria. *J. Mol. Cell. Cardiol.* **46**, 891–901 (2009).

15. Lu, X. *et al.* Measuring Local Gradients of Intramitochondrial [Ca^2+^] in Cardiac Myocytes During Sarcoplasmic Reticulum Ca^2+^ Release. *Circ. Res.* **112**, 424–431 (2012).

16. Haviland, S., Cleemann, L., Kettlewell, S., Smith, G. L. & Morad, M. Diversity of mitochondrial Ca^2+^ signaling in rat neonatal cardiomyocytes: evidence from a genetically directed Ca^2+^ probe, mitycam-E31Q. *Cell Calcium* **56**, 133–46 (2014).

17. Akerboom, J. *et al.* Crystal structures of the GCaMP calcium sensor reveal the mechanism of fluorescence signal change and aid rational design. *J. Biol. Chem.* **284**, 6455–6464 (2009).

18. Henderson, M. J. *et al.* A low affinity GCaMP3 variant (GCaMPer) for imaging the endoplasmic reticulum calcium store. *PLoS One* **10**, e0139273 (2015).

19. Arosio, D. *et al.* Simultaneous intracellular chloride and pH measurements using a GFP-based sensor. *Nat. Methods* **7**, 516–518 (2010).

20. Raimondo, J. *et al.* A genetically-encoded chloride and pH sensor for dissociating ion dynamics in the nervous system. *Front. Cell. Neurosci.* **7**, 202 (2013).

21. Arosio, D. *et al.* Spectroscopic and Structural Study of Proton and Halide Ion Cooperative Binding to GFP. *Biophys. J.* **93**, 232–244 (2007).

22. Miesenböck, G., De Angelis, D. A. & Rothman, J. E. Visualizing secretion and synaptic transmission with pH-sensitive green fluorescent proteins. *Nature* **394**, 192–195 (1998).

23. Thomas, J. A., Buchsbaum, R. N., Zimniak, A. & Racker, E. Intracellular pH measurements in Ehrlich ascites tumor cells utilizing spectroscopic probes generated in situ. *Biochemistry* **18**, 2210–2218 (1979).

24. Brini, M. *et al.* Nuclear Ca2+ concentration measured with specifically targeted recombinant aequorin. *Embo J* **12**, 4813–4819 (1993).

25. Dooley, C. T. *et al.* Imaging dynamic redox changes in mammalian cells with green fluorescent protein indicators. *J. Biol. Chem.* **279**, 22284–22293 (2004).

26. Hanson, G. T. *et al.* Investigating Mitochondrial Redox Potential with Redox-sensitive Green Fluorescent Protein Indicators. *J. Biol. Chem.* **279**, 13044–13053 (2004).

27. Gutscher, M. *et al.* Real-time imaging of the intracellular glutathione redox potential. *Nat. Methods* **5**, 553–559 (2008).

28. Meyer, A. J. & Dick, T. P. Fluorescent protein-based redox probes. *Antioxid. Redox Signal.* **13**, 621–650 (2010).

29. Albrecht, S. C. *et al.* Redesign of genetically encoded biosensors for monitoring mitochondrial redox status in a broad range of model eukaryotes. *J Biomol Screen* **19**, 379–386 (2014).

30. Birk, J. *et al.* Endoplasmic reticulum: reduced and oxidized glutathione revisited. *J. Cell Sci.* **126**, 1604–1617 (2013).

31. Gutscher, M. *et al.* Proximity-based Protein Thiol Oxidation by H_2_O_2_-scavenging Peroxidases. *J. Biol. Chem.* **284**, 31532–31540 (2009).

32. Hung, Y. P., Albeck, J. G., Tantama, M. & Yellen, G. Imaging Cytosolic NADH-NAD+ Redox State with a Genetically Encoded Fluorescent Biosensor. *Cell Metab.* **14**, 545–554 (2011).

33. Hung, Y. P. & Yellen, G. Live-Cell Imaging of Cytosolic NADH–NAD+ Redox State Using a Genetically Encoded Fluorescent Biosensor. in *Fluorescent Protein-Based Biosensors. Methods in Molecular Biology (Methods and Protocols)* (eds. Zhang, J., Ni, Q. & Newman, R.) **1071**, 83–95 (Humana Press, 2014).

34. Tantama, M., Martínez-François, J. R., Mongeon, R. & Yellen, G. Imaging energy status in live cells with a fluorescent biosensor of the intracellular ATP-to-ADP ratio. *Nat. Commun.* **4**, 2550 (2013).

35. Berg, J., Hung, Y. P. & Yellen, G. A genetically encoded fluorescent reporter of ATP:ADP ratio. *Nat. Methods* **6**, 161–166 (2009).

36. Takanaga, H., Chaudhuri, B. & Frommer, W. B. GLUT1 and GLUT9 as major contributors to glucose influx in HepG2 cells identified by a high sensitivity intramolecular FRET glucose sensor. *Biochim. Biophys. Acta - Biomembr.* **1778**, 1091–1099 (2008).

37. Tewson, P. *et al.* Simultaneous detection of Ca^2+^ and diacylglycerol signaling in living cells. *PLoS One* **7**, e42791 (2012).

38. Tewson, P. H., Quinn, A. M. & Hughes, T. E. A multiplexed fluorescent assay for independent second-messenger systems: decoding GPCR activation in living cells. *J. Biomol. Screen.* **18**, 797–806 (2013).

39. Klionsky, D. J. *et al.* Guidelines for the use and interpretation of assays for monitoring autophagy in higher eukaryotes. *Autophagy* **4**, 151–175 (2008).
