# Supplementary material for: Multiplexed Optical Sensors in Arrayed Islands of Cells for multimodal recordings of cellular physiology
Source: Nat Commun. 2020 Aug 4;11:3881. doi: 10.1038/s41467-020-17607-5 (PMC7403318; doi:10.1038/s41467-020-17607-5)
Supplement: Supplementary file 4 — Reporting Summary [file 41467_2020_17607_MOESM4_ESM.pdf]

nature research

Corresponding author(s): Adam E. Cohen

Last updated by author(s): Jun 29, 2020

Reporting Summary

Nature Research wishes to improve the reproducibility of the work that we publish. This form provides structure for consistency and transparency in reporting. For further information on Nature Research policies, see Authors & Referees and the Editorial Policy Checklist.

Statistics

For all statistical analyses, confirm that the following items are present in the figure legend, table legend, main text, or Methods section.

n/a	Confirmed	
☐	☒	The exact sample size (*n*) for each experimental group/condition, given as a discrete number and unit of measurement
☒	☐	A statement on whether measurements were taken from distinct samples or whether the same sample was measured repeatedly
☒	☐	The statistical test(s) used AND whether they are one- or two-sided<br>*Only common tests should be described solely by name; describe more complex techniques in the Methods section.*
☒	☐	A description of all covariates tested
☒	☐	A description of any assumptions or corrections, such as tests of normality and adjustment for multiple comparisons
☐	☒	A full description of the statistical parameters including central tendency (e.g. means) or other basic estimates (e.g. regression coefficient) AND variation (e.g. standard deviation) or associated estimates of uncertainty (e.g. confidence intervals)
☒	☐	For null hypothesis testing, the test statistic (e.g. *F*, *t*, *r*) with confidence intervals, effect sizes, degrees of freedom and *P* value noted<br>*Give P values as exact values whenever suitable.*
☒	☐	For Bayesian analysis, information on the choice of priors and Markov chain Monte Carlo settings
☒	☐	For hierarchical and complex designs, identification of the appropriate level for tests and full reporting of outcomes
☒	☐	Estimates of effect sizes (e.g. Cohen's *d*, Pearson's *r*), indicating how they were calculated

Our web collection on statistics for biologists contains articles on many of the points above.

Software and code

Policy information about availability of computer code

Data collection	Custom instrument control code was written in LabVIEW 2014 to synchronize hardware. Waveforms were generated in MATLAB (R2015a) to synchronously control camera frame acquisition, DMD pattern update, illumination sources, and perfusion valves. Code is available from the corresponding author on reasonable request.
Data analysis	All analyses were performed in MATLAB using custom code. Multiple MATLAB releases were used. Analysis code is compatible with release R2016b. Code is available from the corresponding author on reasonable request.

For manuscripts utilizing custom algorithms or software that are central to the research but not yet described in published literature, software must be made available to editors/reviewers. We strongly encourage code deposition in a community repository (e.g. GitHub). See the Nature Research guidelines for submitting code & software for further information.

Data

Policy information about availability of data

All manuscripts must include a data availability statement. This statement should provide the following information, where applicable:

- Accession codes, unique identifiers, or web links for publicly available datasets
- A list of figures that have associated raw data
- A description of any restrictions on data availability

Data are available from the corresponding author upon reasonable request. The source data underlying Supplementary Figure 23a are provided as a Source Data file.

Field-specific reporting

Please select the one below that is the best fit for your research. If you are not sure, read the appropriate sections before making your selection.

☒ Life sciences ☐ Behavioural & social sciences ☐ Ecological, evolutionary & environmental sciences

For a reference copy of the document with all sections, see nature.com/documents/nr-reporting-summary-flat.pdf

Life sciences study design

All studies must disclose on these points even when the disclosure is negative.

Sample size	Recordings shown are from a single field of view. Within each MOSAIC array, islands were duplicated to control for technical noise, and each island contained > 10 cells. Strong responses of the sensors in response to different perturbations demonstrated the methodology unambiguously. Sample size was not calculated because statistical comparisons between conditions were not part of the study design.
Data exclusions	For all functional MOSAIC recordings, regions of islands were excluded that were contaminated by cells expressing the incorrect sensor, as defined by a quantitative metric. See "Methods > Calculation of fluorescence time traces" for details on the algorithms used.
Replication	We first tested each fluorescent sensor individually, not in the context of a MOSAIC array. In many cases we tested the sensor in both HEK293 cells and cardiomyocytes (See Supplementary figures). We then tested in the context of the full MOSAIC array, which replicated the demonstration of sensor function. All sensors included in Table 1 and Supplementary Table 1 were validated in at least two independent experiment; most in more.<br>Successful MOSAIC measurements were performed on >11 arrays of HEK cells and >11 arrays of cardiomyocytes. Different pharmacological perturbations were made in each case so the data were not aggregated.
Randomization	N/A - no comparison between groups.
Blinding	N/A - no comparison between groups.

Reporting for specific materials, systems and methods

We require information from authors about some types of materials, experimental systems and methods used in many studies. Here, indicate whether each material, system or method listed is relevant to your study. If you are not sure if a list item applies to your research, read the appropriate section before selecting a response.

Materials & experimental systems

n/a	Involved in the study	
☐	☒	Antibodies
☐	☒	Eukaryotic cell lines
☒	☐	Palaeontology
☒	☐	Animals and other organisms
☒	☐	Human research participants
☒	☐	Clinical data

Methods

n/a	Involved in the study	
☒	☐	ChIP-seq
☒	☐	Flow cytometry
☒	☐	MRI-based neuroimaging

Antibodies

Antibodies used	Primary Antibodies:<br>anti-LC3A/B (Rabbit IgG monoclonal, Cell Signaling Technology, Cat. No. 12741, Lot No. 4)<br>anti-GAPDH (Rabbit IgG polyclonal, Sigma-Aldrich, Cat. No. G9545, Lot No. 127M4814V)<br><br>Secondary antibodies:<br>anti-rabbit IgG, HRP-conjugated goat (Abcam, Cat. No. ab6721, Lot No. Gr3192725-5)
Validation	All three antibodies have been well-validated in the literature. According to the vendors, the number of citations for each were: anti-LC3A/B: 347, anti-GAPDH: 937, anti-rabbit IgG: 1928. We confirmed that there are no bands on the western blot if the secondary antibody is applied before the primary antibody. The LC3 and GAPDH antibodies each make a single band on the blot at the anticipated size.

Eukaryotic cell lines

Policy information about cell lines

Cell line source(s)	HEK293T cells: ATCC, #CRL-3216<br>hiPSC-derived cardiomyocytes: Cellular Dynamics International, #iCell cardiomyocytes
Authentication	Cell lines used were purchased directly from vendors and were not further authenticated.
Mycoplasma contamination	Cell lines used in this work were not specifically tested for mycoplasma contamination. Regular mycoplasma testing in the lab has not found evidence of contamination.
Commonly misidentified lines<br>(See ICLAC register)	None used.
