# Supplementary material for: Multiplexed Optical Sensors in Arrayed Islands of Cells for multimodal recordings of cellular physiology
Source: Nat Commun. 2020 Aug 4;11:3881. doi: 10.1038/s41467-020-17607-5 (PMC7403318; doi:10.1038/s41467-020-17607-5)
Supplement: Supplementary file 6 — Description of Additional Supplementary Files [file 41467_2020_17607_MOESM6_ESM.pdf]

Title: Supplementary Video 1. Example MOSAIC recording in HEK cells.
Description: Paired with functional recordings in Fig. 5, a video of 9 selected sensors expressed in HEK cells during metabolic and redox perturbations. For each island pair, the tame-varying background was subtracted using the spatially averaged fluorescence from a frame surrounding the island pair. Movies show $\Delta S/S_0$ calculated according to Table 1. To suppress divide-by-zero noise, the data were masked so only regions with fluorescent cells are shown. Offset and scale for each island were adjusted so that the average value during initial buffer washes is zero, and the magnitude of its maximum deflection is 1. Data are not corrected for pH changes. Some cellular motion is visible during later perfusion steps as cells retract inward.

Title: Supplementary Video 2. Time lapse video of hiPSC-derived cardiomyocytes transduced with the superecliptic pHluorin cytosolic pH sensor.
Description: The lentivirus encoding the fluorescent sensor was spotted with the microarray printer, and the island center-to-center distance was 1 mm. The video spans 48 hours with an image acquired every 5 minutes. Most cells are cardiomyocytes, which show little migration during the period. Highly motile cells are likely not cardiomyocytes.
